# Medicinal plant-derived mtDNA via nanovesicles induces the cGAS-STING pathway to remold tumor-associated macrophages for tumor regression

**DOI:** 10.1186/s12951-023-01835-0

**Published:** 2023-03-06

**Authors:** Jinfeng Liu, Jiaxin Xiang, Cuiyuan Jin, Lusha Ye, Lei Wang, Yanan Gao, Nianyin Lv, Junfeng Zhang, Fuping You, Hongzhi Qiao, Liyun Shi

**Affiliations:** 1 Department of Immunology, School of Medicine and Holistic Integrative Medicine, Nanjing University of Chinese Medicine, Nanjing, 210023 Jiangsu China; 2 Institute of Translational Medicine, Zhejiang Shuren University, Hangzhou, 310015 Zhejiang China; 3 Institute of Systems Biomedicine, Department of Immunology, School of Basic Medical Sciences, Beijing Key Laboratory of Tumor Systems Biology, NHC Key Laboratory of Medical Immunology, Peking University Health Science Center, Beijing, 100191 China; 4 Jiangsu Key Laboratory for Functional Substance of Chinese Medicine, Jiangsu Engineering Research Center for Efficient Delivery System of TCM, School of Pharmacy, Nanjing University of Chinese Medicine, Nanjing, 210023 China

**Keywords:** Artemisia-derived nanovesicles, Tumor-associated macrophages, mtDNA, cGAS-STING

## Abstract

Plant-derived nanovesicles (PDNVs) have been proposed as a major mechanism for the inter-kingdom interaction and communication, but the effector components enclosed in the vesicles and the mechanisms involved are largely unknown. The plant *Artemisia annua* is known as an anti-malaria agent that also exhibits a wide range of biological activities including the immunoregulatory and anti-tumor properties with the mechanisms to be further addressed. Here, we isolated and purified the exosome-like particles from *A. annua*, which were characterized by nano-scaled and membrane-bound shape and hence termed artemisia-derived nanovesicles (ADNVs). Remarkably, the vesicles demonstrated to inhibit tumor growth and boost anti-tumor immunity in a mouse model of lung cancer, primarily through remolding the tumor microenvironment and reprogramming tumor-associated macrophages (TAMs). We identified plant-derived mitochondrial DNA (mtDNA), upon internalized into TAMs via the vesicles, as a major effector molecule to induce the cGAS-STING pathway driving the shift of pro-tumor macrophages to anti-tumor phenotype. Furthermore, our data showed that administration of ADNVs greatly improved the efficacy of PD-L1 inhibitor, a prototypic immune checkpoint inhibitor, in tumor-bearing mice. Together, the present study, for the first time, to our knowledge, unravels an inter-kingdom interaction wherein the medical plant-derived mtDNA, via the nanovesicles, induces the immunostimulatory signaling in mammalian immune cells for resetting anti-tumor immunity and promoting tumor eradication.

**Graphical Abstract:**

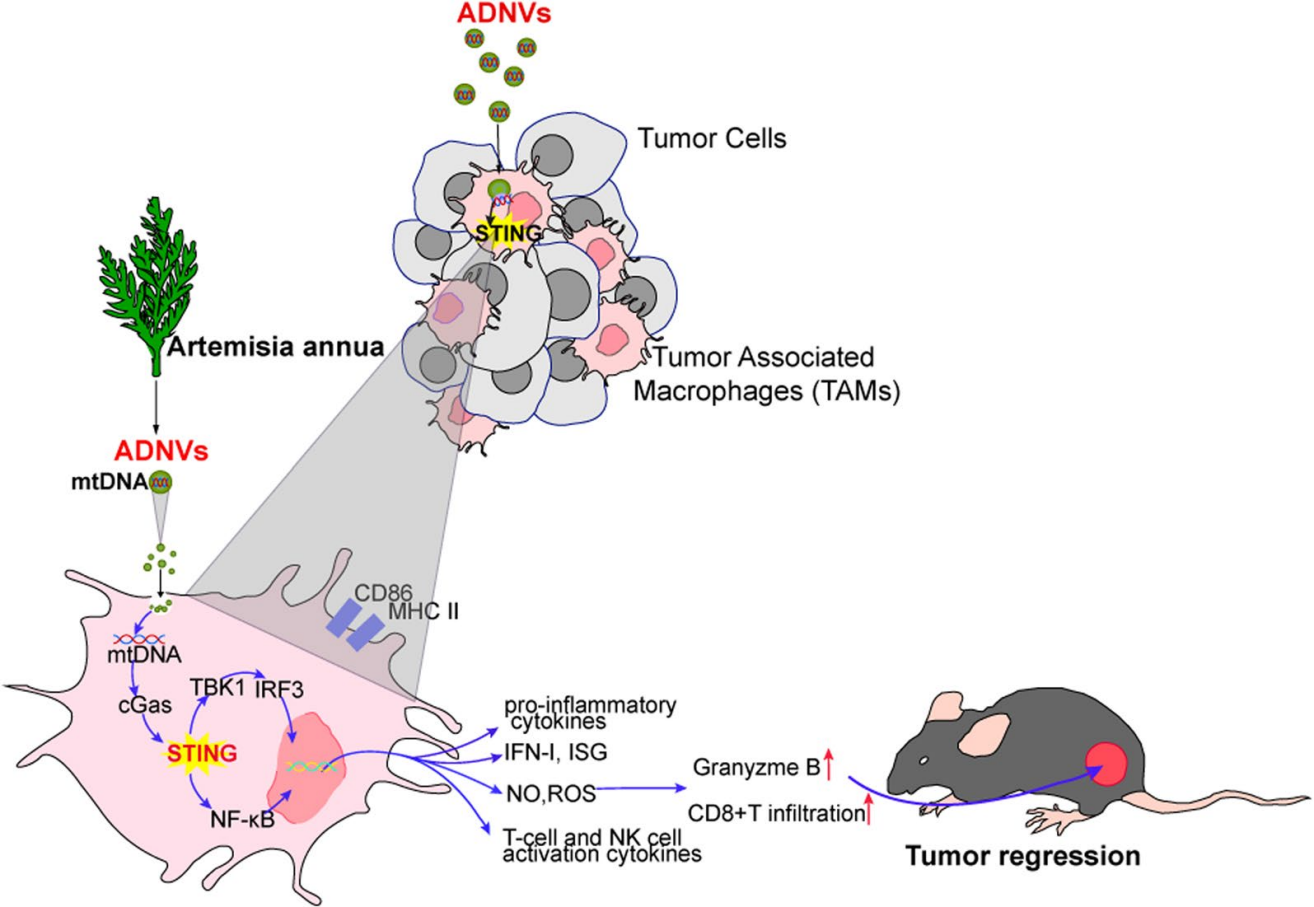

**Supplementary Information:**

The online version contains supplementary material available at 10.1186/s12951-023-01835-0.

## Introduction

Inter-kingdom communication and interactions has been documented to occur between eukaryotic and prokaryotic cells. Accumulating evidences demonstrate that the tissues and cells from humans, plants, animals or even microorganism release chemical substances or cellular components to trigger various signaling pathways, profoundly affecting the phenotype and function of recipient cells. However, current works are mostly focused on the human-microorganism interaction, and little is known about how plant-originated factors are interconnected with mammal cells. Indeed, emerging evidences have indicated that, akin to animal cells, plant cells can automatically or passively release nano-scaled vesicles, known as plant-derived nanovesicles (PDNVs), to induce the specific pathways and impact pathophysiology of targeting cells [1–3]. PDNVs contain a range of cellular components including RNA, DNA, lipids, proteins and secondary metabolites, which may act as the signaling messengers to mediate the signaling transduction and gene expression regulation [4], thereby exerting multiple effects involving anti-inflammatory, anti-viral, anti-fibrotic and anti-tumor activities [5–9]. For instances, the nanovesicles from plants like Ginseng were recently reported to potentially imped cancer growth and improve the immunotherapy efficacy, presumably through increasing the infiltration of immune cells [10]. However, it remains to be further determined about the effector components in the vesicles and the molecular underpinnings of PDNV-mediated immunoregulation.

Artemisinin is a sesquiterpene endoperoxide known as a frontline treatment against Plasmodium falciparum malaria. The agent is extracted from the plant *Artemisia annua* by a team of Chinese scientists led by Youyou Tu, who received the Nobel Prize in 2015 for discovering the anti-malaria drug [11]. Notably, recent data demonstrated that, in addition to canonical anti-parasite activity, artemisinin and the relevant medicinal plant could be repurposed to treat the inflammatory diseases, viral infections, fibrosis, autoimmune diseases and cancer [12, 13]. Of particular interest, artemisinin and its derivatives were found to potentially modulate the differentiation, activation and function of immune cells such as T cells, B cells, macrophages and dendritic cells, making it a potential agent to modify tumor microenvironment and anti-tumor immunity [14–16], but the technical restraints such as limited output, low biocompatibility, and little knowledge of the mechanism hamper their further application as potent antitumor or immunoregulatory agents.

Recent study demonstrate that the cyclic GMP-AMP synthase (cGAS)-stimulator of interferon genes (STING) pathway plays a key role in regulating immune and inflammatory response, and may serve as an important mechanism to regulate tumor progression [17]. cGAS is generated upon recognizing intracellular dsDNA and induces the second messenger, cyclic GMP-AMP (cGAMP), which in turn activates the key signaling adaptor STING. The activated STING subsequently triggers the downstream NF-κB and IRF3-driven pathways, leading to the production of inflammatory cytokines, chemokines and IFN-β [18] to induce immune cell infiltration and activation. Considering that progressive tumors are generally associated with impaired immune cell activation, coined as “cold” status, the cGAS-STING pathway may become a promising way to “wake up” the tolerant immune cells such as tumor-associated macrophages (TAMs). Intense research is therefore undertaken to search for natural and synthesized STING ligands/activators to improve the efficacy of cancer therapy [19]. Cyclic dinucleotides (CDNs), for instance, are one of the best characterized STING agonists. Several flaws such as highly negatively charge, difficulty to cross cell membranes, vulnerability to enzymatic degradation preclude the agent however to be an ideal anti-tumor agent. Compared with this, PDNVs are generally wrapped with lipophilic membrane, accessible for target cells, and importantly, capable of delivering nuclear acids to induce the immune-sensing machinery like STING pathway in recipient cells. In this regard, studies from us and other investigators have revealed that exosome-like vesicles harboring the cellular components such as mitochondrial DNA (mtDNA) would be secreted by mammal cells and taken by the recipients to induce STING pathway [20–22]. In addition to animal cells, DNA or cGAMP from prokaryotic cells like viruses or bacteria can also be incorporated into the membrane vesicles, triggering the STING pathway for the inter-kingdom regulation [23, 24]. In plants, the horizontal transfer of mitochondria or mtDNA has been reported to occur between the species [25–27], but whether the DNA-containing vesicles are generated by the medicinal plant like *Artemisia annua*, and how the encapsulated components signal the immune cells to regulate the anti-tumor immunity have never been explored.

In this study, we isolated and purified the nano-scaled vesicles from *Artemisia annua*. The newly discovered vesicles, termed ADNVs, displayed a robust anti-tumor activity through reprogramming TAMs from pro-tumor phenotype to pro-inflammatory type. Mechanistically, artemisia-derived mtDNA were taken by TAMs via the vesicles and induced the cGAS-STING pathway to reprogram macrophages, leading to the enhanced cytotoxic T response for tumors regression. We further demonstrate that ADNVs improved the efficacy of αPD-L1-mediated immunotherapy through the activation of STING-driven pathway. To our knowledge, it’s the first time to report that nanovesicle-harboring, plant-derived mtDNA mediates the inter-kingdom communication for resetting the anti-tumor immunity.

## Materials and methods

### Animals and cell lines

Pathogen-free, 6-to-8-week-old male C57BL/6 mice were obtained from Jiangsu Gempharmatech Co., Ltd (Jiangsu, China). STING knockout mice (STING^gt/gt^ mouse, 017537) were used and genotyped according to the protocols provided by The Jackson Laboratory. The mice were raised routinely: Temperature 22–25 °C, relative humidity 50–60%, and a 12 h light–dark cycle. All animals were given food and water in a standard laboratory diet. All animal experiments were reviewed and approved by the Institutional Animal Care and Use Committee (IACUC) of Nanjing University of Chinese Medicine (Approval No. 202207A023).

LLC cell line, mouse colon cancer cell line (CT26), human embryonic kidney cell line (HEK293T) and RAW 264.7 were obtained from American Type Culture Collection (ATCC, USA). All cell lines were cultured in Dulbecco’s Modified Eagle’s Medium (DMEM) or RPMI 1640 with 10% fetal bovine serum (FBS), supplemented with 100 U/mL penicillin, and 100 mg/mL streptomycin (all from Gibco, Carlsbad, CA, USA). All cells were incubated at 37 °C in a humidified atmosphere with 5% CO2.

### Isolation and purification of ADNVs

For isolation of ADNVs, fresh *Artemisia annua* L. was washed three time with water in a plastic bucket. After the final wash, the plant in phosphate-buffered saline (PBS) (1:2, g/mL) were placed in a blender and chopped at a high speed for 5 min. The obtained juices were sequentially centrifuged at 200 × g for 10 min, 2000 × g for 20 min and 10,000 × g for 30 min to remove the large plant tissues and cell debris. The final supernatant was ultracentrifuged at 150,000 × g for 2 h, and the pellets were re-suspended in PBS. To further purify ADNVs, their suspension was transferred to a gradient sucrose solution (15%, 30%, 45% and 60%), and ultracentrifuged at 150,000 × g for 2 h. Finally, ADNVs in the 30% layer were harvested and washed 3 times with PBS. The resuspension was filtered (0.45 μm) and used freshly or stored at − 80 °C until further use. The amounts of ADNVs were quantified on the basis of their protein amounts, which were determined using a BCA protein assay kit.

### Tumor growth and treatment

C57BL/6 mice were maintained in an animal facility under pathogen-free conditions. After acclimatization, a total of 2 × 10^5^ LLC cells were subcutaneously injected into the right flank of the mice. Tumor dimensions were measured with calipers every 3 days and the tumor volumes (mm^3^) were calculated by applying the following formula: (length × width^2^)/2. ADNVs (25 mg/kg), alone or with αPD-L1 antibody (10 mg/kg) (Bio-XCell, BP0101), were instilled at day 7 after tumor cell inoculation, every 3 days for 2 weeks. To deplete TAMs in tumor, LLC tumor-bearing mice were treated with clodronate liposomes (CL, Yeasen, 40337ES08) at 200 μg per mouse every 4 days by intraperitoneal injection. Mice in the control group were treated with the same dose of liposomes containing PBS (PL, Yeasen, 40338ES05). On the indicated days after inoculation, mice were sacrificed, and the tissues were collected for analysis.

### ADNVs labeling and ex vivo organ imaging

ADNVs labeling with Dil was performed according to the manufacturer’s instructions (ThermoFisher, D282). In brief, 200 mg of ADNVs were suspended in 1 mM Dil staining solution and incubated for 30 min at room temperature. ADNVs were pelleted by ultracentrifuge at 150,000 × g for 2 h to remove free dye. After washing twice in PBS, Dil-labeled ADNVs were ready for use in experiments.

For ex vivo organ imaging, the mice were intraperitoneally injected with Dil-labeled ADNVs (25 mg/kg), and samples were collected for detection 24 h later. The organs were resected and imaged on an Odyssey scanner (LI-COR, USA).

### Tumor digestion and TAMs isolation

To obtain single-cell suspensions, tumors were excised, minced and digested with 5% FBS DMEM containing 2 mg/mL collagenase I (Gibco, 17100017) and 2 mg/mL hyaluronidase (Sigma-Aldrich, H6254) at 37 °C for 45 min with agitation, followed by treatment with ammonium-chloride-potassium buffer for red blood cell (RBC) lysis, and strained through a 70 μm strainer to remove undigested tumor tissues.

For TAMs isolation, the single-cell suspensions were centrifuged at 500 × g for 20 min, with 1 mL of the cell suspension on the top, 5 mL of 45% Percoll (GE Healthcare, 17-0891-09) in the middle and 5 mL of 60% Percoll at the bottom of a 15-mL tube. Mononuclear cells were collected from the cell layer in the interphase between 45 and 60% Percoll. F4/80^+^ TAMs were isolated using anti-F4/80 Microbeads (Miltenyi Biotec, 130-110-443) according to the manufacturer’s instructions.

### Mouse bone marrow-derived macrophages (BMDMs) preparation and polarization

Bone marrow was harvested from 8 to 10 weeks old mice by flushing the femurs with PBS. Following RBC lysis, the remaining cells were washed twice with PBS. For induction of macrophage differentiation, the cells were cultured in DMEM supplemented with 10% FBS and 20 ng/mL mouse macrophage colony-stimulating factors (M-CSF) (PeproTech, 315-02). After 3 day of culture, cell media were replenished with the media containing 20 ng/mL M-CSF. On day 7, M2 polarized macrophages were obtained by treatment with mouse 20 ng/mL IL-4 (PeproTech, 214-14) for 48 h. After polarization, cells were phenotyped and used in subsequent assays.

### Flow cytometry and FACS sorting

Tumor cell suspensions, BMDMs, RAW 264.7and TAMs were washed, blocked with Fc Block (anti-mouse CD16/32, BD Biosciences) at 4 °C for 15 min, and stained with fluorescence-conjugated antibodies against surface markers CD4 (clone OKT4), CD8 (clone 17D8), F4/80 (clone BM8), CD11b (clone VIM12), CD86 (clone BU63), CD206 (clone MR5D3) and PD-L1 (clone MIH5) antibodies purchased from BioLegend or BD Biosciences. Cells were then fixed in a fixation/permeabilization buffer (BD Biosciences, 554715) and stained with antibodies against intracellular proteins, including Granzyme B (clone GB11, BioLegend). The apoptosis of cells were detected using Annexin V-FITC/PI Apoptosis Detection Kit (BD Biosciences, 556547). Single cell suspensions were prepared and analyzed using the FACS Calibur (BD Biosciences). FACS-sorted CD206^+^ macrophages are from BMDMs for adoptive transfer experiments by using a BD FACS Aria-II SORP flow cytometer (BD Biosciences). Data analysis was performed using FlowJo Version7.6 (BD Biosciences).

### Adoptive transfer experiments

For analysis of the role of TAMs in ADNVs antitumor, 1 × 10^5^ CD206^+^ macrophages were sorted from BMDMs of mice with or without ADNVs treatment, and adoptively transferred into the tumor-bearing mice by intratumoral injection every 3 days initiating 14 days after tumor cell inoculation. On 21 day after inoculation, tumor-bearing mice were anesthetized, and the tissues were collected for analysis.

For analysis of the effect of ADNVs in promoting TAMs polarization, 1 × 10^5^ CD206^+^ macrophages sorted from BMDMs were labeled with Dil, and adoptively transferred into tumor-bearing mice by intratumoral injection. Mice were then administered intraperitoneally with or without ADNVs for 24 h, and the tumor tissues were collected for analysis.

### ROS measurement

To measure cellular ROS levels, cells were stained with CellROX Green (ThermoFisher, C10444), which generates fluorescent signals when oxidized by ROS in the cells. The mean fluorescence intensity (MFI) of ROS was detected by flow cytometry and analyzed using FlowJo Version7.6 (BD Biosciences).

### ELISA

Cell culture supernatants were harvested and subjected to centrifugation at 500 × g for 10 min at 4 °C to remove floating cells and debris. The levels of TNF-α and IL-6 were detected via mouse TNF-α ELISA Kit (R&D, MTA00B) and mouse IL-6 ELISA Kit (R&D, M6000B) according to manufacturer’s instructions.

### Quantitative real-time PCR (qRT-PCR)

Total RNA was extracted from macrophages or tumor tissues using TRIzol reagent (Invitrogen, 15596026) and reverse transcribed into cDNA using a cDNA Synthesis Kit (Takara, 6210A) according to the manufacturer’s instructions. Then, qRT-PCR was performed using SYBR Green mix (Roche, 4913914001) following the manufacturer’s instructions. Samples were run on an ABI Prism 7500 Sequence Detection System (Applied Biosystems, USA). The primers used for target genes were shown in Additional file 1: Table S1. The 2^−ΔΔCt^ method was used to calculate fold changes in gene expression normalized to β-actin.

### Western blotting

Protein was extracted from the cells using RIPA buffer, resolved by SDS–polyacrylamide gels and then transferred to PVDF membranes. Primary antibodies against STING (1:1000; CST, 13647), Phospho-TBK1 (1:1000; CST, 5483), TBK1 (1:1000; CST, 38066), Phospho-IRF-3 (1:1000; CST, 29047), IRF-3 (1:1000; CST, 4302), Phospho-p65 (1:1000; CST, 3033), p65 (1:1000; CST, 8242), GAPDH (1:1000; CST, 5174) and β-actin (1:1000; CST, 4970) were used. Peroxidase-conjugated secondary antibody (CST, 7077/7076) was used, and the antigen–antibody reaction was visualized by enhanced chemiluminescence assay (ECL, ThermoFisher, 34580).

### Immunofluorescence and immunohistochemistry

Paraffin-embedded samples were sectioned at 4-mm thickness. Antigen retrieval was performed using a pressure cooker for 3 min in 0.01 M citrate buffer (pH 6.0). Samples were blocked in PBS with 2% BSA for 1 h at room temperature and incubated with antibodies specific for Ki-67 (1:100; Abcam, ab16667), MMP9 (1:100; Abcam, ab283575), CD86 (1:100; Abcam, ab119857), CD206 (1:100; Abcam, ab64693), iNOS (1:100; Abcam, ab178945) and Arg1 (1:100; Abcam, ab96183) overnight at 4 °C. Alexa Fluor or HRP conjugated secondary antibodies was incubated for 1 h at room temperature. DAPI was then used for counterstaining the nuclei, and images were obtained by fluorescence microscope (Axio Observer D1, Zeiss).

### Genomic DNA isolation and PCR

ADNVs were collected and their genomic DNA was isolated using DNeasy Blood & Tissue Kits (Qiagen, 69504). DNA in the nuclear, chloroplast and mitochondria was identified by PCR (Takara, R010A) following the manufacturer’s instructions. The primer used for target genes were shown in Additional file 1: Table S2. The products were separated by electrophoresis on a 1% agarose gel stained with ethidium bromide (EB).

### MtDNA depletion

ADNVs were treated with EtBr (200 ng/mL) for 6 days to deplete mtDNA from the vesicles. The mtDNA depletion efficiency was evaluated by PCR test of total DNA extraction. The primers were provided in Additional file 1: Table S2.

### Statistical analysis

The values are presented as the mean ± standard error of mean (SEM). All data were analyzed using GraphPad Prism 8 (GraphPad Software, USA). The data were examined using the two-tailed Student’s *t* test, one-way analysis of variance (ANOVA), or two-way ANOVA with post hoc Bonferroni correction. *p* < 0.05 was considered statistically significant (*p < 0.05, **p < 0.01, ***p < 0.001).

## Results

### Purification and characterization of ADNVs

**Fig. 1:**
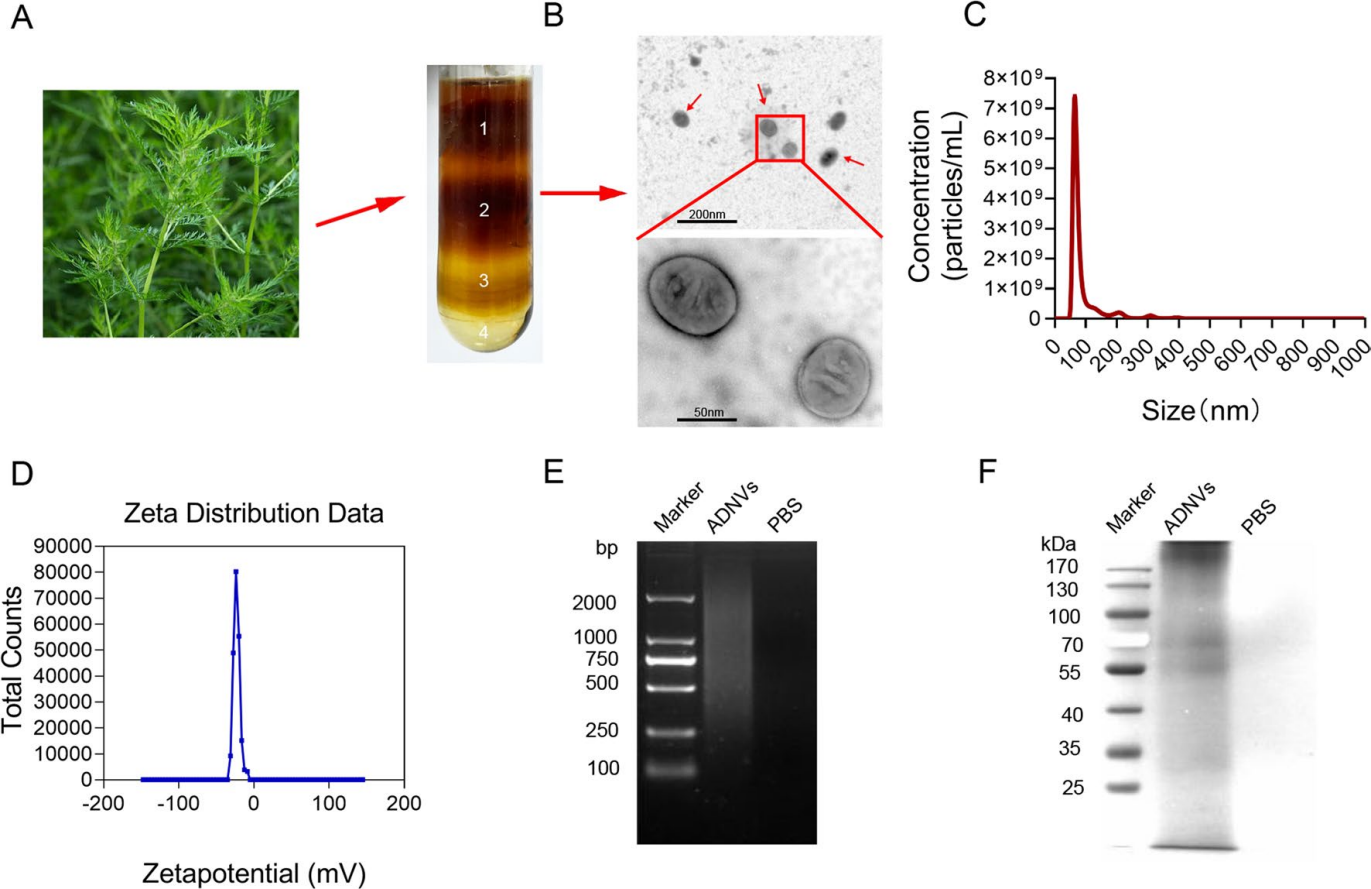
Purification and characterization of ADNVs. **A** ADNVs were isolated and purified by differential the centrifugation and sucrose gradient ultracentrifugation. **B** ADNVs harvested from the sucrose density gradient (30%) were characterized by TEM (Scale bar = 200/50 nm). **C** Particle size of the ADNVs was measured by NanoSight NS 300 system. **D** Surface charge was measured by a Zetasizer. **E** ADNVs-enclosed DNA was electrophoresis on a 1.2% agarose gel, and stained with EtBr. **F** Proteins of ADNVs were separated by 10% SDS-PAGE and stained with Coomassie blue. Shown are the representative results from at least three independent experiments

To explore the biological activity of Artemisia-derived micro-vesicles, we isolated and purified exosome-like vesicles from fresh *Artemisia annua* L. by exploiting a method combining differential centrifugation and sucrose density gradient ultracentrifugation. As a result, four bands were observed after the sucrose density gradient ultracentrifugation (Fig. 1a), with the majority accumulated at the 30% interface (band 2). Transmission electron microscopy (TEM) revealed that the vesicles were characterized by spherical morphology and bilayer membrane structure (Fig. 1b). An average hydrodynamic size of ADNVs was 106.8 nm as detected by NanoSight NS 300 system, and the concentration was about 1.25 × 10^11^ vesicles per mL in solution (Fig. 1c). Zeta potential analysis indicated that ADNVs had a negative zeta potential value of − 22.5 mV (Fig. 1d). In addition, we analyzed the composition of purified ADNVs. The results showed that substantial amounts of nucleic acids and proteins were encapsulated in ADNVs, as examined by agarose gel electrophoresis and SDS-PAGE electrophoresis respectively (Fig. 1e, f).

Next, we examined the impact of ADNVs on cellular *via*bility using cell counting kit-8 (CCK-8) and lactate dehydrogenase (LDH) assays respectively. The results demonstrated that, up to the dose of 160 μg/mL, ADNVs exhibited no significant cytotoxicity on murine macrophages (Additional file 1: Fig. S1A, B). To further evaluate the in vivo safety of ADNVs, we treated mice with ADNVs at the dose up to 25 mg/kg via intraperitoneal (i.p.) injection. Two weeks later, mice were sacrificed for blood biochemistry analysis and histological examinations. The results showed that no apparent damages were observed in the heart, liver, spleen, lung, kidney, or intestines in mice upon ADNVs administration (Additional file 1: Fig. S1C). Lower rather than higher levels of liver enzymes such as alanine transaminase (ALT) and aspartate transaminase (AST) were detected in ADNVs-treated mice relative to control animals, suggesting no significant liver toxicity associated with this treatment (Additional file 1: Fig. S1D, E). Together, our data indicated that the nanovesicles were successfully isolated from *Artemisia annua*, which exhibited no toxic effects in vitro and in vivo at the doses we applied at later experiments.

### ADNVs treatment impedes cancer growth in a murine lung cancer model

**Fig. 2:**
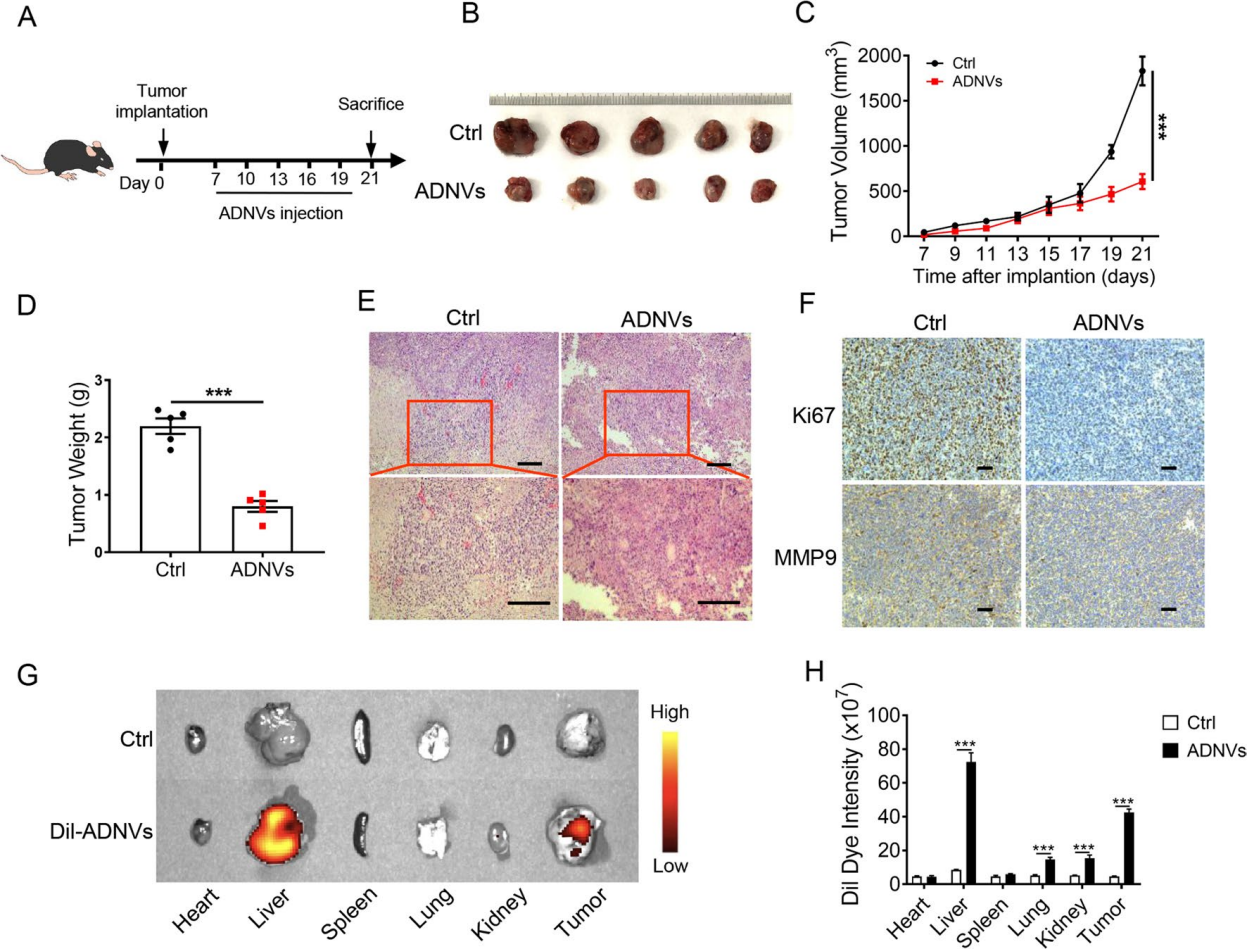
ADNVs inhibit lung cancer growth in mice. **A** The simplified experimental scheme. C57BL/6 mice (n = 5) were implanted with LLC cells for 7 d, and then treated with ADNVs (25 mg/kg, i.p.) once every 3 d for a successive 2 week. Mice were sacrificed and tumors were collected at day 21. **B** Gross photos of tumors at the end of experiments. **C** Tumor growth profiles in tumor-bearing mice treated PBS or ADNVs. ***p < 0.001 (Two-way ANOVA and Bonferroni post-tests). **D** Tumor weights in mice treated with either PBS or ADNVs. ***p < 0.001 (Student’s *t*-test). **E** H & E staining of tumor tissues (Scale bar = 100 μm). **F** Ki67 and MMP9 staining of tumor tissues (Scale bar = 100 μm). (G, H) ADNVs were stained with Dil and injected into tumor-bearing mice (25 mg/kg, i.p.). Biodistribution of ADNVs was determined by scanning mice (**G**), and the quantitative analysis (**H)**. ***p < 0.001 (One-way ANOVA and Tukey’s significant difference post hoc test). The results are representative data from one of three independent experiments. Shown are representative images, and the data are presented as means ± SEM

To evaluate the potential role of ADNVs in curbing the common malignancy such as lung cancer, we established a murine lung cancer model by subcutaneously inoculating lewis lung carcinoma (LLC) cells into C57BL/6 mice. After 7 days, mice were treated with ADNVs or vehicle once every 3 days for successive 2 weeks (Fig. 2a). Remarkably, ADNVs administration led to a profound inhibition of tumor growth, as evidenced by smaller sizes, reduced tumor volume and weights in the vesicle-treated group relative to control mice (Fig. 2b–d). Tumors in treated mice exhibited a lower cellular density with nuclear pyknosis, suggestive of cellular apoptosis (Fig. 2e). The levels of Ki67 and MMP9, the molecular marker for cellular proliferation and metastasis respectively, were decreased in ADNVs-treated mice relative to control animals (Fig. 2f).

To further confirm the action of ADNVs in tumors, we next examined the in vivo trafficking of ADNVs. For this, the vesicles were pre-stained with a lipophilic fluorescent dye, Dil, and then administrated into the tumor-bearing mice at 3 weeks post inoculation. The Odyssey imaging analysis revealed that, in addition to liver enrichment, Dil-ADNVs were dominantly concentrated in tumor tissues (Fig. 2g, h). In addition, we compared the efficacy of different routes, such as intraperitoneal (i.p.), intravenous (i.v.), subcutaneous (s.c.) and intratumoral (i.t.) administration to deliver ADNVs to tumors. The results showed that, compared with i.p. injection, the route of both i.t. and i.v. rendered ADNVs to efficiently reach the tumor site, while s.c. injection exhibited negligible effect (Additional file 1: Fig. S2A, B). Moreover, ADNVs instillation via i.t. and i.v. had similar effects in tumor control compared to that via i.p. injection, while s.c. injection had much smaller effect (Additional file 1: Fig. S2C–E). Together, the data demonstrated that ADNVs treatment significantly inhibited tumor growth, impairing the malignant properties involving cellular proliferation, invasion and apoptosis-resistance in tumors.

### ADNVs treatment remolds tumor microenvironment and promotes macrophages shift to pro-inflammatory phenotype

**Fig. 3:**
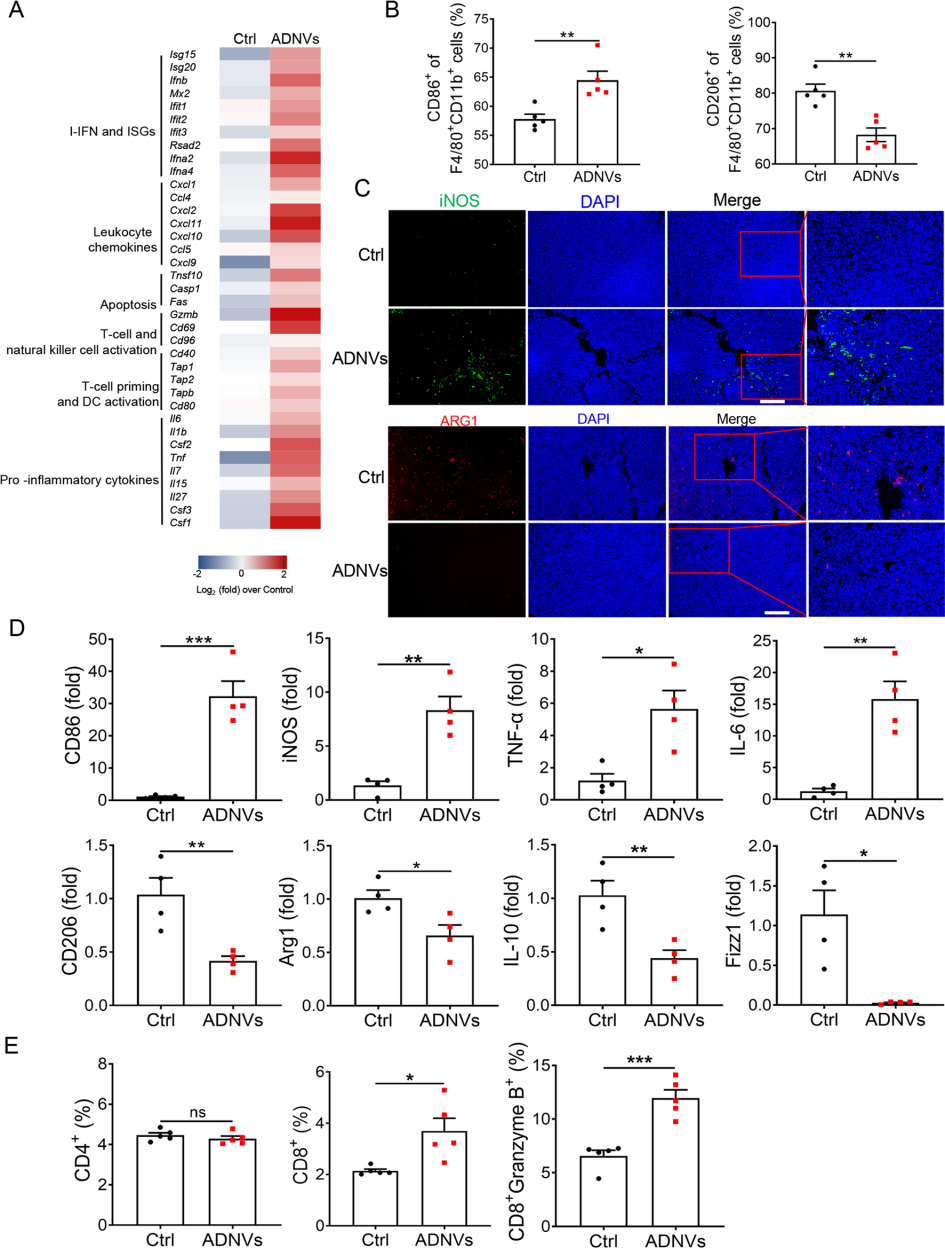
ADNVs remold tumor microenvironment and reprogram TAM phenotypes. Mice were implanted with LLC and treated with ADNVs or vesicle as described in Fig. 2. Tumor tissues were collected at 21 d post inoculation. **A** Ranked analysis of differential gene expression. **B** Quantification of M1 (CD86^+^) and M2 (CD206^+^) populations by flow cytometry. **p < 0.01 (Student’s *t*-test). **C** Representative immunofluorescence staining for CD86 and CD206 at tumor sections (Scale bar = 100 μm). **D** qRT-PCR analysis of M1-marker genes (upper) and M2-related genes. *p < 0.05, **p < 0.01, ***p < 0.001 (Student’s *t*-test). **E** Quantification of CD4^+^, CD8^+^ and Granzyme B^+^ CD8^+^ cell populations in TME by flow cytometry. *ns* non-significant, *p < 0.05, ***p < 0.001 (Student’s *t*-test). The results are from one of three independent experiments. Shown are representative images, and the data are presented as means ± SEM

Since the properties of tumor microenvironment (TME), immune-tolerant (“cold”) or immunogenic (“hot”) [28], have a determinative role in cancer initiation and progression, we therefore proceeded to assess the impact of ADNVs on the properties of TME. Critically, ADNVs treatment caused a remarkable shift of TME to an inflamed and tumor-unfavorable status, characterized by highly expression of pro-inflammatory cytokines, leukocyte-recruiting chemokines, type I interferon (IFN) and IFN-stimulated genes (ISGs), as well as the signature genes related with dendritic cell maturation and T-cell priming (Fig. 3a).

It is well appreciated that TAMs, predominately populating in TME, would undergo functional reprogramming from pro-inflammatory M1 to pro-tumor M2 phenotype and support cancer progression [29]. We thus investigated the impact of ADNVs on the phenotypic shift of TAMs. Remarkably, ADNVs treatment elevated the ratio of M1-like type macrophages (defined by CD86^+^CD11b^+^F4/80^+^) while repressing the portion of M2-like subset (defined by CD206^+^CD11b^+^F4/80^+^) (Fig. 3b, Additional file 1: Fig. S3A). Consistently, the percentage of MHC-II^+^ macrophages was increased while that of PD-L1^+^ cells was decreased in TAMs from ADNVs-treated mice (Additional file 1: Fig. S3B, C), as compared with those in vehicle-treated animals. The M2/M1-modulating role of ADNVs was further confirmed by immunofluorescence (IF) staining of iNOS (M1) and ARG1 (M2) at tumor tissues (Fig. 3c). Congruently, the transcriptional profiling of TAMs revealed that ADNVs treatment strengthened the expression of M1-related markers but repressed that of M2-associated genes (Fig. 3d). The data thus consistently supported the role of ADNVs in promoting macrophages polarization from pro-tumor type to anti-tumor subset.

Macrophages are known as professional antigen presenting cells (APCs) with the ability to process and present antigens, produce inflammatory cytokines and chemokines for stimulating robust T cell response. In line with augmented M1 polarization induced by ADNVs, we observed that the frequency of intratumoral CD8^+^ T cells, particularly granzyme B^+^ T cells, was markedly increased in ADNVs-treated mice relative to control animals (Fig. 3e, Additional file 1: Fig. S3D). No significant change was detected in the ratio of CD4^+^ T cells between the two groups of mice. Collectively, our data demonstrated that ADNVs treatment profoundly changed the immunological profile of TME, promoting the transition of TAMs to elevate cytotoxic T lymphocytes (CTLs) response, and thereby expediting the eradication of lung cancer.

### TAMs were targeted by ADNVs to mediate the anti-tumor effect

**Fig. 4:**
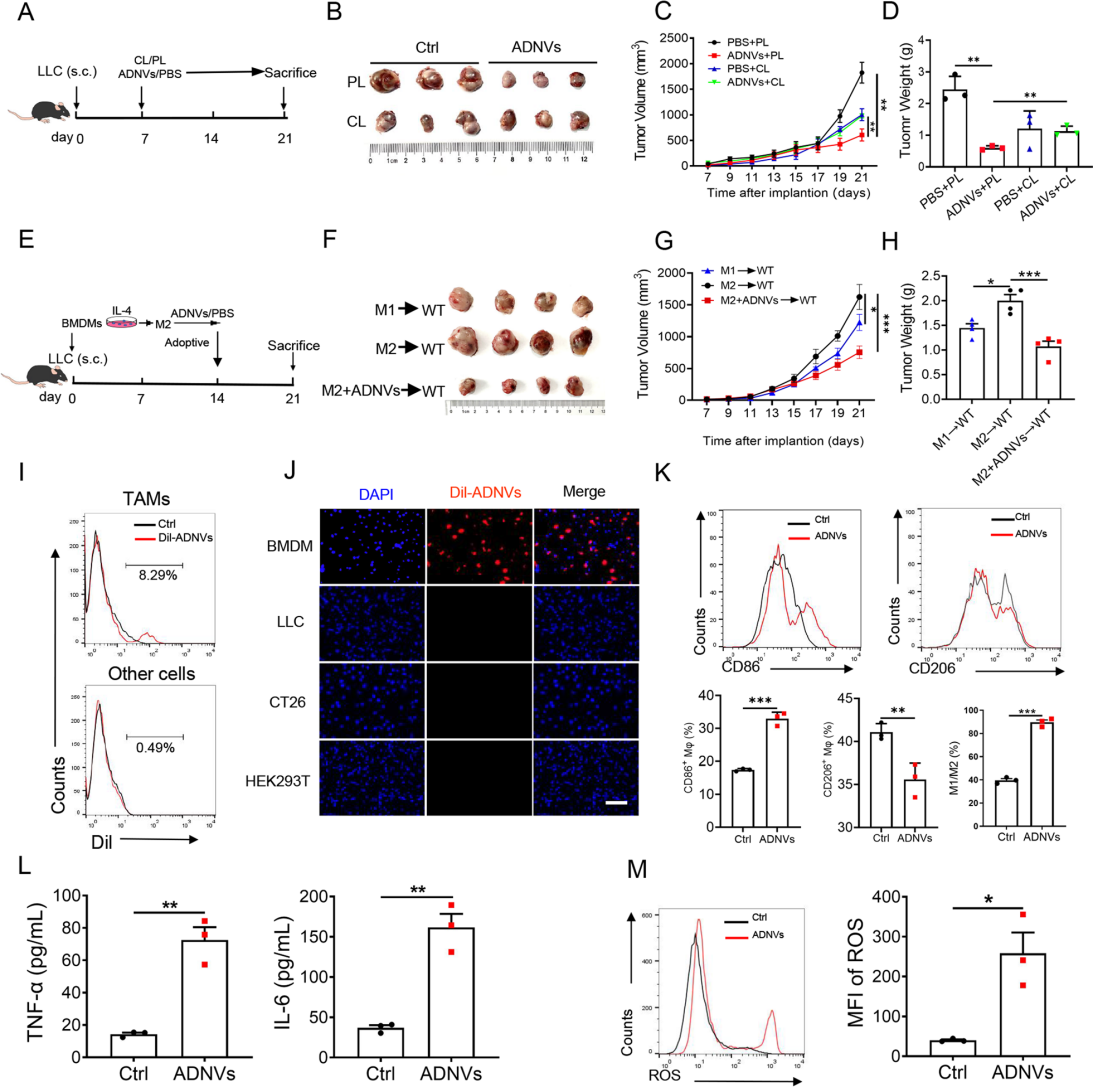
ADNVs are preferentially taken by TAMs and reprogram their phenotypes. **A** The simplified experimental scheme for B-D. C57BL/6 J mice were implanted with LLC cells for 7 d and then inoculated with clodronate liposomes (CL) or PBS-liposomes (PL) every 4 days to deplete TAMs. The mice were simultaneously administrated with ADNVs, and sacrificed at day 21 post implantation. **B** Gross photos of tumors at the end of experiments. **C** Tumor growth profiles. **p < 0.01 (Two-way ANOVA and Bonferroni post-tests). **D** Tumor weights at the end of the experiment. **p < 0.01 (One-way ANOVA and Tukey’s significant difference post hoc test). **E** Schematic outline of adoptive transfer of macrophages for F–H. M2 macrophages were prepared from BMDMs by stimulated with IL-4, and then treated with or without ADNVs (20 μg/mL). M1 macrophages, prepared by stimulation of IFN-ɣ, were used as a positive control. After that, the cells were adoptively transferred to tumor-bearing mice at day 14 post LLCs implantation. Mice were sacrificed at day 21. **F** Gross photos of tumors at the end of experiments. **G** Tumor growth profiles. *p < 0.05, ***p < 0.001 (Two-way ANOVA and Bonferroni post-tests). **H** Tumor weights at the end of the experiment. *p < 0.05, ***p < 0.001 (One-way ANOVA and Tukey’s significant difference post hoc test). **I**, **J** ADNVs were stained with Dil and injected into tumor-bearing mice (25 mg/kg, i.p.). Flow cytometry of Dil^pos^ cells in macrophages and other cell populations in tumors (**I**). Immunofluorescence (IF) staining of BMDMs and other cell lines taking Dil-labelled ADNVs upon incubation. Scale bar = 200 μm. Nuclear: DAPI (**J**). (K-M) BMDMs were incubated with ADNVs or vehicles for 24 h. Quantification of M1 (CD86^+^) and M2 (CD206^+^) population by flow cytometry (**K**); ELISA assay of TNF-α and IL-6 levels (**L**); Flow cytometry of ROS^pos^ macrophages and quantification of the mean fluorescence intensity (MFI) (**M**). *p < 0.05, **p < 0.01, ***p < 0.001 (Student’s *t*-test). The results are from one of two or three independent experiments. Shown are representative images, and the data are presented as means ± SEM

To determine whether TAMs was responsible for ADNVs-mediated tumor growth inhibition, we then applied clodronate liposome (CL) to deplete TAMs from mice that were implanted with LLCs and treated with ADNVs at the first week (Fig. 4a). As expected, TAMs were efficiently cleared upon CL treatment while administration of the control liposome (PL) had slight effect (Additional file 1: Fig. S4A). Depletion of TAMs remarkably decreased tumor burdens in mice with no ADNVs administration, implying the pro-tumor activity of TAMs in this context. By contrast, CL administration significantly increased tumor growth in ADNVs-instilled mice compared with PL treatment, indicating that the vesicle-remolded macrophages exerted the cancer inhibitory effect (Fig. 4b–d).

To further corroborate the relevance of macrophages to ADNVs action in tumor control, we then applied the adoptive transferring methods. To this end, M2-polarized macrophages, which were generally induced by growing tumors, were prepared by incubating murine bone marrow-derived macrophages (BMDMs) with IL-4, the canonical stimulators for type II immune cells. The pre-fabricated cells were then conditioned with ADNVs prior to the delivery to tumor-bearing mice (Fig. 4e). The data showed that the mice taking ADNVs-primed macrophages developed significantly smaller tumors compared with those receiving vehicle-treated macrophages (Fig. 4f–h), further supporting a pivotal role of macrophages in mediating the tumor-inhibiting activity of ADNVs.

As the active contents of natural nanoparticles exert their biological functions mainly within cells, we wondered whether ADNVs could traffic to the tumors and exert biological their regulatory effects on TAMs. Flow cytometry analysis of Dil-ADNVs was thus conducted on TAMs and other remaining cells isolated from tumors. The results indicated that ADNVs trafficked to the tumors and were dominantly taken up by TAMs, when compared with the fluorescent signals detected in other remaining cells (Fig. 4i, Additional file 1: Fig. S3E). In vitro, BMDM, LLC, CT26 and HEK293T cells were incubated with Dil-ADNVs for 12 h. Compared to other cells, we found that ADNVs were taken up more effectively by BMDMs and preferentially localized in the cytoplasm of the cells (Fig. 4j). The results thus indicated the cellular tropism of ADNVs to macrophages in vitro and in vivo.

Next, we examined the impact of ADNVs on macrophages function. Remarkably, ADNVs treatment significantly raised the ratio of CD86^+^ M1 cells, while decreasing the proportion of CD206^+^ M2 cells in cell culture (Fig. 4k). Concurrently, production of the proinflammatory cytokines TNF-α and IL-6 was elevated by ADNVs-treated pre-made M2 macrophages compared with that by vehicle-treated cells (Fig. 4l), indicating the functional shift of pre-made M2 cells induced by ADNVs treatment. Given that M1 macrophages are capable of killing tumor cells by producing the effector mediators such as reactive oxygen species (ROS), we thus further measured the impact of ADNVs on macrophages generation of ROS. The data showed that the percentage of ROS-producing macrophages was substantially increased upon ADNVs treatment (Fig. 4m). Associated with this, the apoptotic rate of lung cancer cells, as detected by Annexin V/PI staining or caspase-3 activity, was markedly elevated upon incubation at the culture medium (CM) from ADNVs-treated macrophages relative to that from vehicle-treated cells (Additional file 1: Fig. S4B, C). Collectively, the data indicated that ADNVs trafficked to tumors and preferentially taken up by TAMs, leading to the reprogramming of TAMs into a proinflammatory phenotype for a “hot” (immunostimulatory) tumor microenvironment.

### ADNVs reprogram TAMs through activating the cGAS-STING pathway

**Fig. 5:**
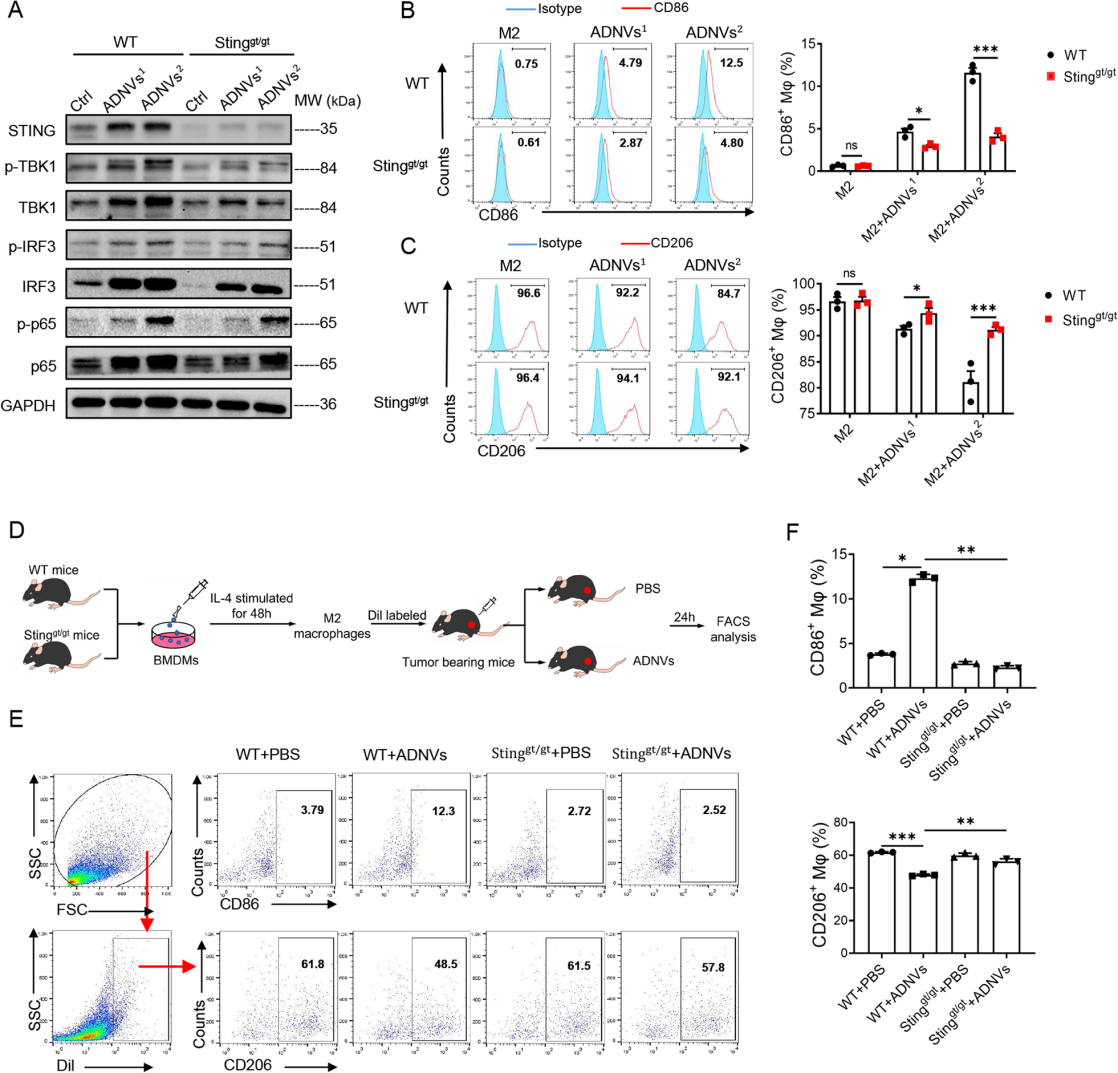
ADNVs promote the functional transition of TAMs through activation of the STING pathway. BMDMs from WT mice and STING^gt/gt^ mice were polarized into M2 type and then treated with two doses of ADNVs^1^ (20 μg/mL) or ADNVs^2^ (30 μg/mL) for 24 h. **A** Immunoblotting of STING and downstream signaling molecules in macrophages. **B**, **C** Flow cytometry analysis and quantification of M1 (CD86^+^) and M2 (CD206^+^) populations. ns: non-significant, *p < 0.05, ***p < 0.001 (Student’s *t*-test). **D** Diagram of workflow for the in vivo experiments. BMDMs from WT mice and STING^gt/gt^ mice were polarized into M2 type, labeled with Dil, and transferred into tumor-bearing mice. The animals were then treated with ADNVs for 24 h. **E** Flow cytometry and **F** quantification of the portion of M1 (CD86^+^) and M2 (CD206^+^) subsets respectively. *p < 0.05, **p < 0.01, ***p < 0.001 (One-way ANOVA and Tukey’s significant difference post hoc test). The results are from one of three independent experiments. Shown are representative images, and the data are presented as means ± SEM

We next sought to address the mechanism that was exploited by ADNVs to regulate macrophages phenotypes and functions in tumors. Our and other investigators’ studies have shown that donor cells would be able to transfer cellular components like mtDNA to the recipients and induce the inflammatory signaling involving the GAS-STING pathway [21, 22]. The induction of STING pathway activates downstream IRF3 and TBK1 to drive the inflammatory cytokines and IFN-I production, thereby disrupting the immune tolerant status in tumor niches [30–32]. Based on these premises, we hypothesized that ADNVs might induce the cGAS-STING pathway to promote the polarization of inflammatory macrophages that were pre-conditioned by tumors. Indeed, our initial study showed that the expression of type I IFN and associated ISG genes were specifically induced by ADNVs in TAMs (Fig. 3a). In line with this, the activation of STING and the downstream signaling molecules involving TBK1, IRF3 and p65 in pre-fabricated M2 macrophages was remarkably increased upon ADNVs treatment (Fig. 5a). The regulatory function of ADNVs was largely abrogated in macrophages from STING-knockout goldenticket (gt) mice, and the subsequent M1/M2 macrophage shift was accordingly impeded (Fig. 5b, c). The results thus corroborated the specific activation of STING pathway by ADNVs administration, which consequentially induced the M2 to M1 functional transition.

Inspired by the above findings, we further assessed the in vivo relevance of STING pathway to the action mode of ADNVs. To this end, BMDMs from STING^gt/gt^ and control mice were pre-induced into M2 type, stained with Dil, and adoptively transferred into LLC-bearing mice (Fig. 5d). After administration with ADNVs but not the vehicle, we observed that a portion of Dil-labeled macrophages in tumors shifted from CD206^+^ M2 to CD86^+^ M1 subset. This transition however did not occur in STING^gt/gt^ macrophages regardless of ADNVs administration (Fig. 5e, f). Collectively, the results indicated that ADNVs administration specifically induced the activation of STING pathway to promote the transition of TAMs from M2 to M1 phenotype and hence enhance anti-tumor immunity.

We also investigated the role of other immune pathways such as TLRs in the antitumor activity of ADNVs. We additionally conducted the experiments with macrophages lacking some of TLRs such as TLR2, TLR3 and TLR4 respectively. (Additional file 1: Fig. S5A–C) The results showed that deletion of TLR2, TLR3 or TLR4 exerted mild or even no impact on the pro-inflammatory and M1-promoting activity of ADNVs, indicating that TLRs pathway are unlikely the major contributors for ADNVs function.

### Medicinal plant-derived mtDNA encapsulated in ADNVs triggers the activation of STING pathway in TAMs

**Fig. 6:**
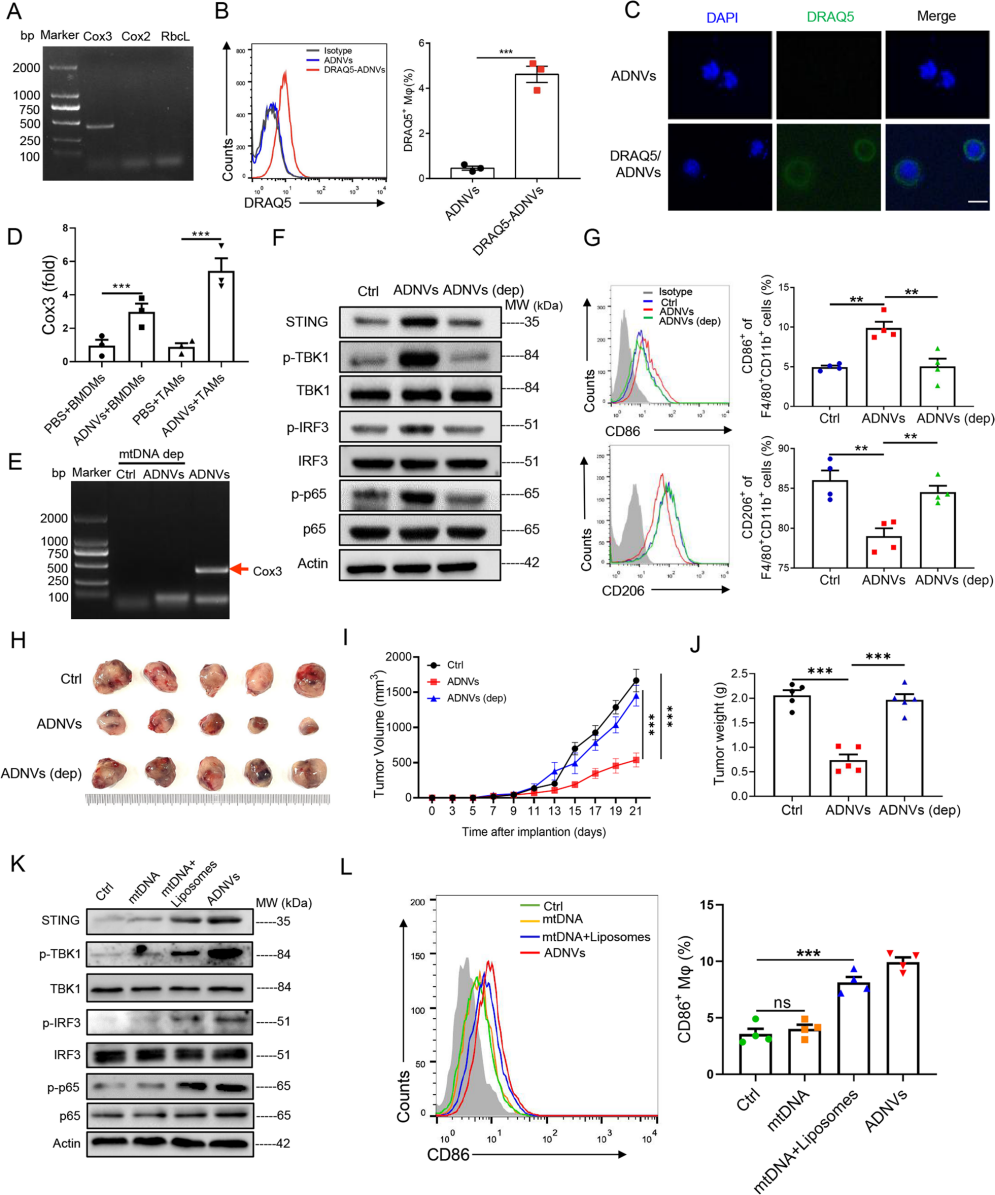
Plant-derived mtDNA activates STING pathway to drive macrophage polarization. **A** PCR assay of the genes *Cox3*, *Cox2* and *RbcL* in ADNVs. **B**, **C** ADNVs were stained by DRAQ5 and incubated with BMDMs for 6 h. Flow cytometry and quantification of the portion of DRAQ5^+^ cell population (***p < 0.001, Student’s *t*-test) **B**, and immunofluorescence staining showing mtDNA-taking of DRAQ5^+^ cells. Nuclei: DAPI; Scale bar = 50 μm (**C**). **D** qRT-PCR analysis of *Cox3* gene expression in BMDMs treated with ADNVs or vehicle, or in TAMs isolated from mice that were administrated with ADNVs or PBS. ***p < 0.001 (One-way ANOVA and Tukey’s significant difference post hoc test). **E**–**G** ADNVs were treated with or without EtBr (200 ng/mL) for 6 d to deplete mtDNA, and then applied to M2-polarized macrophages. PCR assay of *Cox3* gene in ADNVs (**E**); Immunoblotting of STING and downstream signaling molecules (**F**); Flow cytometry and quantification of the percentages of M1 (CD86^+^) and M2 (CD206^+^) populations (**G**). **p < 0.01 (One-way ANOVA and Tukey’s significant difference post hoc test). **H**–**J** ADNVs were treated with or without EtBr to deplete mtDNA, and then injected into LLC-bearing mice (25 mg/kg, i.p.). **H** Gross photos of tumors at the end of experiments (21 day post LLCs inoculation. **I** Tumor growth profiles. ***p < 0.001 (Two-way ANOVA and Bonferroni post-tests). **J** Tumor weights evaluated at the end of the experiment. ***p < 0.001 (One-way ANOVA and Tukey’s significant difference post hoc test). **K**, **L** mtDNA was extracted from ADNVs, coated with or without liposomes and then applied to M2-polarized macrophages for 24 h. Immunoblotting of STING and downstream signaling molecules (**K**); Flow cytometry and quantification of the percentage of M1 (CD86^+^) populations (**L**). ns: non-significant, ***p < 0.001 (One-way ANOVA and Tukey’s significant difference post hoc test). The results are from one of two or three independent experiments. Representative images are shown and the data are presented as means ± SEM

Considering the well-appreciated role of STING pathway in recognizing cytosolic dsDNA and inducing the subsequent immunostimulatory pathway [33], we hypothesized that plant-derived dsDNA might be delivered by vesicles to trigger STING pathway in macrophages. To test it, we firstly analyzed the DNA content in ADNVs by PCR analysis of plant-specific genes including *Cox3* (housekeeping gene for mitochondrial DNA), *Cox2* (housekeeping gene for nuclear DNA) and *Rbcl* (housekeeping gene for chloroplast DNA). Intriguingly, the results showed that only *Cox3*, a representative mtDNA gene, was amplified in ADNVs (Fig. 6a). To directly confirm the transferring of mtDNA via the nanovesicles, we labeled ADNVs with DRAQ5, a membrane-permeable DNA dye [34], and added them onto pre-made M2 macrophages. After complete washing, the intracellular fluoresce intensity of macrophages was measured. Flow cytometry revealed that mtDNA-containing vesicles were prominently taken by macrophages (Fig. 6b), and the internalization of vesicular DNA in macrophages was further confirmed by IF staining (Fig. 6c). To further confirm that mtDNA was effectively internalized by macrophages in tumors, we additionally isolated TAMs and performed the qRT-PCR analysis of *Cox3*. Similar to the in vitro result, significantly greater amount of *Cox3* was detected in TAMs from ADNVs-treated mice, but not in those from vehicle-treated control mice (Fig. 6d). The data thus indicated that medicinal plant-derived mtDNA was encapsulated in the nanovesicles for delivering to macrophages in tumors.

To further substantiate the functional significance of mtDNA in the regulatory role of ADNVs, we treated the vesicle with ethidium bromide (EtBr), an agent presumed to selectively deplete mtDNA without affecting genomic DNA [35]. The vesicles were then applied to pre-made M2 macrophages for the analysis of STING activation. As expected, EtBr treatment eliminated mtDNA from the vesicles, as demonstrated by compromised amplification of the representative gene, *Cox3* (Fig. 6e). The activation of STING-driven pathway was substantially weakened (Fig. 6f), and the M2 to M1 shift was largely abrogated upon mtDNA depletion (Fig. 6g). As a result, the mice treated with mtDNA-depleted ADNVs developed significantly greater tumors compared with those taking the intact vesicles (Fig. 6h–j).

The results indicated that medicinal plant-derived mtDNA in the nanovesicles played a major role in inducing the anti-tumor immunity, making us to ask whether naked mtDNA or mtDNA enclosed by the vesicle membrane exerted the effect. To address it, we extracted mtDNA from ADNVs and constructed the mtDNA/lipid complex using synthetic liposomes, which were then added onto pre-made M2 macrophages to test their effects. Of interest, the data showed that naked mtDNA failed to induce M1 macrophages polarization, while lipid-encapsulated mtDNA promoted CD86^+^ macrophages generation, at the level comparable to that of ADNVs treatment (Fig. 6k, l). Together, our data identified plant-derived mtDNA as a critical stimulus for STING activation when internalized into macrophages via nanovesicles, and played an essential role in inducing anti-tumor immunity.

### ADNVs treatment boosts the immunotherapeutic efficacy of PD-L1 blockade in mice

**Fig. 7:**
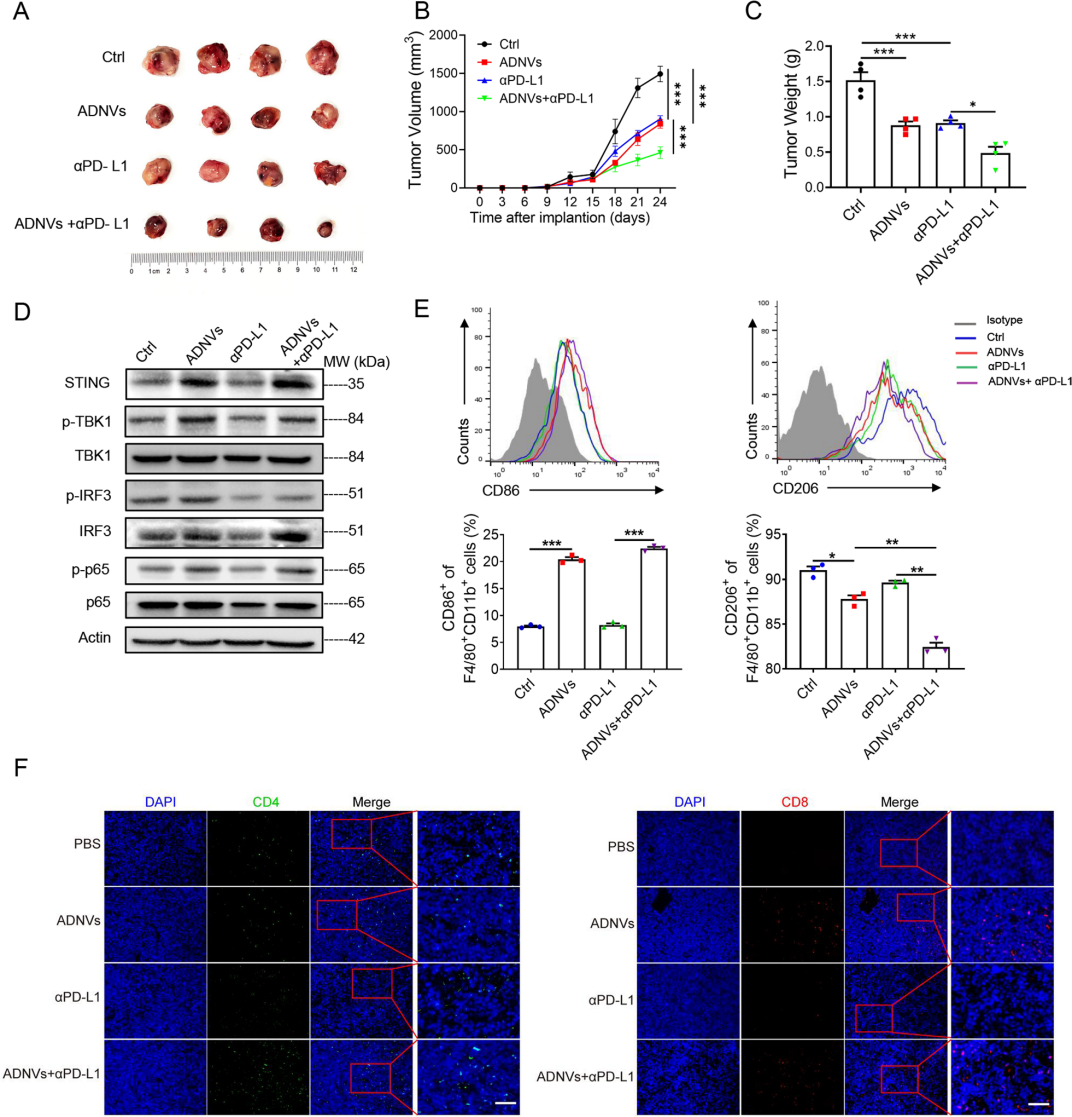
ADNVs strengthen the immunotherapy efficacy of PD-L1 blockade in mice. C57BL/6 mice were implanted with LLC cells for 7 days, and then instilled with ADNVs (25 mg/kg), alone or with αPD-L1 antibody, every 3 days for 2 weeks. Tumors were collected at day 21 post-implantation. **A** Gross photos of tumors at the end of experiments. **B** Tumor growth profiles in mice. ***p < 0.001 (Two-way ANOVA and Bonferroni post-tests). **C** Tumor weights at the end of the experiment. *p < 0.05, ***p < 0.001 (One-way ANOVA and Tukey’s significant difference post hoc test). **D** Immunoblotting of STING and downstream signaling molecules in TAMs; **E** Flow cytometry and quantification of the portions of M1 (CD86^+^) and M2 (CD206^+^) population in TAMs. *p < 0.05, **p < 0.01, ***p < 0.001 (One-way ANOVA and Tukey’s significant difference post hoc test). **F** Immunofluorescence staining of CD4^+^ and CD8^+^ cells in tumors. Nuclei: DAPI; Scale bar = 50 μm. The results are from one of two independent experiments. Representative images are shown and the data are presented as means ± SEM

As the above observation demonstrated that ADNVs potentially induced the transition of TAMs from M2 to M1 phenotype, and the expression of PD-L1 was decreased in ADNVs-treated macrophages (Additional file 1: Fig. S3C), we speculated that ADNVs might enhance the therapeutic efficacy of PD-L1 blockade, a frontline immune checkpoint therapy [36]. To test it, combinative treatment of ADNVs and αPD-L1 was applied in a murine lung cancer model. The results demonstrated that, compared with the treatment of αPD-L1 alone, combination of ADNVs and αPD-L1 elicited a more pronounced effect in impeding tumor growth (Fig. 7a–c). In parallel, the activation of STING-driven pathway was enhanced in TAMs upon co-administration of ADNVs and αPD-L1. Macrophage polarization from M2 to M1 was consistently boosted (Fig. 7d, e), and the numbers of CD4^+^ and CD8^+^ cells were elevated in mice co-instilled with ADNVs and αPD-L1, when compared with those in mice receiving αPD-L1 monotherapy (Fig. 7f). The data thus indicated that ADNVs administration, through STING-mediated macrophage reprogramming, greatly improved the efficacy of checkpoint blockade therapy.

## Discussion

Plant-derived nanovesicles (PDNVs) are membranous vesicles, with lipid bilayers as the basic framework to encapsulate active substances such as proteins, lipids, and nucleic acids [37, 38]. Evidences have demonstrated that PDNVs can serve as a major pathway to mediate material exchange and information transfer between species, thereby playing a pivotal role in regulating cell function, tissue repair, and self-defense [39, 40]. PDNVs, mostly derived from edible plants or herbs, have been reported to exert multiple effects including anti-inflammatory, anti-viral, anti-fibrosis and anti-tumor activity. Known for their well-described features such as safety, biocompatability and few side effects, PDNVs are increasingly recognized a promising therapeutic agents or drug carriers to treat the disorders like cancer, infection and autoimmune diseases [8, 41, 42]. However, the key issues, such as the bioactive contents enclosed in the vesicles, the targeted cells, and the signaling pathway induced, remain to be addressed. In this study, we isolate and purify artemisia-derived nanovesicles and establish them as a potential anti-tumor agent via reprogramming macrophages and hence anti-tumor immunity. Importantly, our data demonstrate that medicinal plant-derive mtDNA, enclosed in the nanovesicles and preferentially taken by TAMs, induces the cGAS-STING pathway to promote the transition of macrophages from the immune-tolerant to proinflammatory phenotypes, and thereby controls tumor progression. It’s the first time, to our knowledge, to describe a cross-kingdom interaction wherein plant-derived mtDNA impacts the phenotype and function of TAMs to rewire the anti-tumor immunity in mammals. Also, we identify an unappreciated role for the cGAS-STING pathway in mediating the immunoregulatory activity of plant-derived nanovesicles, opening an avenue for developing new anti-tumor strategies, alone or combination with αPD-L1 (Fig. 7).

Artemisinin has been recognized as a potent and effective antimalarial drug that has been widely applied in the world [43]. Beyond anti-parasite activity, artemisinin and its derivatives exhibit the therapeutic potentials for viral infections, inflammatory disease, autoimmune diseases and cancer [44–47]. Also, the extracts of *Artemisia annua*, particularly artemisinin and flavonoids, have been reported to have numerous pharmacological properties [48, 49]. However, we still know little about the action mode of Artemisinin and the relevant components. Recent data have demonstrated that nanovesicle from medicinal or edible plants like grape, grapefruit, ginger, and ginseng, have multiple advantages including rich resources, low immunological risk, low cost efficiency and readiness for mass production, making them a promising therapeutics for relevant diseases [3]. In this study, we purified the nano-scaled vesicles from *Artemisia annua* by the ultracentrifugation combined with density gradient centrifugation. The electron microscopy and zeta potential analyses reveal that the vesicles have the structure similar to mammalian-derived exosomes. These properties may help ADNVs to overcome the biological barriers to effectively deliver the bioactive materials such as protein, lipids and nucleic acids to recipient cells. In particular, our data identify that the enclosed mtDNA served as a major effector molecule to mediate the immunoregulatory function of ADNVs. Since most of current PDNVs studies are focused on microRNAs (miRNAs) [5, 7, 50], our discovery of mtDNA and its biological activities provides a novel insight into the action mode of plant-derived nanovesicles. To confirm the entity and action of mtDNA in vesicles, we carefully screen and exclude the possible sources of contaminations. Firstly, plant-specific mitochondrial DNA, but not genomic and chloroplast DNA, were amplified in the purified vesicles. This amplifi cation was abolished upon mtDNA depletion, further confirming the mtDNA contents in the vesicles; Secondly, mtDNA encapsulated in the vesicle, labeled by the lipophilic dye Dil, can be evidently detected in recipient cells, supporting its origin of donor cells not recipients themselves; Thirdly, enzymatic digestion of nucleic acid or depletion of mtDNA led to the abolishment of STING activation and macrophages polarization; and importantly, pretreatment of mtDNA eliminator largely abrogated the inhibitory effect of ADNVs in tumor growth, bolstering the immunoregulatory role for mtDNA in the nanovesicles. Interestingly, our comparative analysis reveals that naked mtDNA, with no lipid-dominant membrane encapsulated, lost the ability to induce STING pathway when added onto macrophages. The data is reminiscent of previous report that administration of the agents by means of nanovesicles is more efficient, although the action of the enclosed materials is the same, whether they are free or encapsulated [51]. Studies have shown that natural STING ligands such as cyclic dinucleotides (CDNs) are hydrophilic, negatively charged, hard to cross cell membranes, and susceptible to enzymatic degradation. Lipid nanoparticles enhance the stability of CDNs, and allow them to be more efficiently taken up by the phagocytes, and to penetrate deep and accumulate into tumors [52]. For these reasons, lipid nanodisc can be exploited to deliver a range of therapeutic cargos to treat tumors [42, 53]. Future studies might be required to further clarify the lipid components of Artemisia-derived nanovesicles and dissect their roles as the delivery vector or even the immune stimulatory agents.

TAMs are the most abundant immune cells in tumor microenvironment, playing a pro-tumor or anti-tumor role depending on the phenotypes they adopted. Upon exposure of proinflammatory stimuli, macrophages undergo the functional transition to immunogenic M1 phenotype and induce the antitumor immunity by releasing the immunostimulatory cytokines such as IL-1β, IL-12 and IL-23, reactive nitrogen and oxygen inter mediates. By contrast, macrophages in rapidly growing tumors tend to adopt alternatively activated M2 phenotype, with release of IL-10 and expression of mannose and arginase1 (Arg1), resistin-like α (Fizz1), and chitinase-like 3 (Ym1), thereby promoting tumor cell proliferation, metastasis and angiogenesis [54]. The high plasticity of macrophages makes them an attractive target to be manipulated for cancer immunotherapy, and the agents with macrophage-modifying properties have a therapeutic potential for cancers. Our data demonstrate that intraperitoneal instillation of ADNVs efficiently trafficked to tumor tissues, preferentially taken up by intratumoral macrophages. The data are in line with previous studies indicating macrophage-targeting property of plant-derived vesicles [41, 55]. This is likely due to the potential of macrophages to uptake and internalize nanoparticles primarily through receptor-mediated internalization and membrane-fusing machinery. Our recent study revealed the requirement of clathrin and caveolae-mediated endocytosis for internalization of exosome-like vesicles [20]. Future studies might be merited to clarify the mechanism responsible for the internalization of PDNVs and their cellular preference.

Current studies have identified many mechanisms driving macrophage reprogramming and the shift of TME from “cold” to “hot” status. Ligands for the innate immune sensors such as toll-like receptors (TLRs), retinoic acid-inducible gene I (RIG-I), nucleotide-binding oligomerization domain (NOD) have been reported to potentially enhance anti-tumor immunity. The GAS-STING pathway, upon recognizing cytosolic dsDNA, initiates IRF3 and NF-κB-driven pathways to induce proinflammatory macrophages polarization and strengthen anti-tumor immunity [56, 57]. Recent studies have shown that leakage of dsDNA from dying tumor cells would reprogram TAMs from pro-tumor M2 type to an anti-tumor M1 phenotype through activating STING pathway [31, 58]. The pathway was mitigated by phagocytic clearance of apoptotic tumor cells [33], indicating that the stability and persistence of STING ligands are required for optimized anti-tumor response. Our present studies identify plant-derived mtDNA as an unappreciated ligand for STING in rewiring macrophage and subsequent CTL response. Compared with the traditional ligands for STING like cyclic dinucleotides (CDNs), vesicular mtDNA exhibits a variety of merits such as lipophilic property, membrane permeability and resistance to degradation by nucleases. Additionally, as artemisinin and medicinal plants have been widely applied in clinic for years, their safety, biocomparability and in vivo dynamics are guaranteed. All the traits make ADNVs a promising therapy or drug carriers for treating tumors or other related illness [59, 60].

Of particular interest, our data also show that ADNVs treatment significantly enhanced the efficacy of PD-1 blockade in a mice lung cancer model. Although the mechanism for this synergism is to be further addressed, it was recently shown that the STING pathway exerted the regulatory role by transcriptional regulating the production of reactive oxygen species (ROS), which may enhance cellular apoptosis to reduce cancer burden and simultaneously functions as an immunogenic stimuli [61, 62]. In agreement with this concept, our data show that ADNVs treatment markedly elevated the percentage of ROS-generating macrophages, accompanied by increased apoptosis of cancer cells and augmented anti-tumor T cell response. This leads to the speculation that ADNVs may also modulate cellular ROS homeostasis to induce immunogenic cell death, thereby bridging the innate and adaptive immunity to coordinate anti-tumor response. The findings raise a rationale for developing ADNVs-based therapeutics, either alone or combination with other immunotherapy or traditional cancer treatments such as chemotherapy and radiotherapy, which can potentially induce immunogenic cell death [63, 64].

## Conclusions

In summary, we purified and characterized the nanovesicles from *Artemisia annua* and revealed its anti-tumor potential through remolding tumor microenvironment and reprogramming macrophages. The cGAS-STING pathway, induced by plant-derived mtDNA, was identifies as a major mechanism for the immunoregulatory role of ADNVs. We thus uncover an unexplored inter-kingdom interaction between the medicinal plant and mammal immune systems, which might be exploited to develop new treatments for tumors.

## Supplementary Information

**Additional file 1: Fig. S1**. Analysis of biocompatibility of ADNVs. **Fig. S2**. Effects of different injection routes on biodistribution and tumor control of ADNVs in vivo. **Fig. S3**. ADNVs promote TAMs polarization towards M1 phenotype. **Fig. S4**. ADNVs act through TAMs to exert the anti-tumor effect. **Fig. S5**. The immunoregulatory function of ADNVs is not through TLR2, TLR3, or TLR4 pathway. Tablse S1. Mouse primers for quantitative RT-PCR analysis. **Table S2**. Plant primers for PCR analysis.

## Data Availability

The data that support the findings of this study are available from the corresponding authors upon reasonable request.

## Abbreviations

ADNVs: Artemisia-derived nanovesicles
ALT: Alanine transaminase
ANOVA: One-way analysis of variance
APCs: Antigen presenting cells
Arg1: Galactose receptors arginase1
AST: Aspartate transaminase
ATCC: American Type Culture Collection
BMDMs: Bone marrow-derived macrophages
CDNs: Cyclic dinucleotides
cGAMP: Cyclic GMP-AMP
cGAS: Cyclic GMP-AMP synthase
CL: Clodronate liposome
CM: Culture medium
CT26: Mouse colon cancer cell line
CTLs: Cytotoxic T lymphocytes
DMEM: Dulbecco’s Modified Eagle’s Medium
EB: Ethidium bromide
FBS: Fetal bovine serum
Fizz1: Resistin-like-α
gt: Golden ticket
HEK293T: Human embryonic kidney cell line
IACUC: Institutional Animal Care and Use Committee
IF: Immunofluorescence
IFN: Type I interferon
ISGs: IFN-stimulated genes
LDH: Lactate dehydrogenase
LLC: Lewis lung carcinoma
MFI: Mean fluorescence intensity
miRNAs: MicroRNAs
mtDNA: Mitochondrial DNA
NOD: Nucleotide-binding oligomerization domain
PBS: Phosphate-buffered saline
PDNVs: Plant-derived nanovesicles
PL: Control liposome
RIG-I: Retinoic acid-inducible gene I
ROS: Reactive oxygen species
SEM: Standard error of mean
STING: Stimulator of interferon genes
TAMs: Tumor associated macrophages
TEM: Transmission electron microscopy
TLRs: Toll-like receptors
TME: Tumor microenvironment
Ym1: Chitinase-like 3
αPD-L1: Anti-PD-L1
