# Supplementary material for: Medicinal plant-derived mtDNA via nanovesicles induces the cGAS-STING pathway to remold tumor-associated macrophages for tumor regression
Source: J Nanobiotechnology. 2023 Mar 6;21:78. doi: 10.1186/s12951-023-01835-0 (PMC9990354; doi:10.1186/s12951-023-01835-0)
Supplement: Supplementary file 1 — Additional file 1: Fig. S1. Analysis of biocompatibility of ADNVs. Fig. S2. Effects of different injection routes on biodistribution and tumor control of ADNVs in vivo. Fig. S3. ADNVs promote TAMs polarization towards M1 phenotype. Fig. S4. ADNVs act through TAMs to exert the anti-tumor effect. Fig. S5. The immunoregulatory function of ADNVs is not through TLR2, TLR3, or TLR4 pathway. Tablse S1. Mouse primers for quantitative RT-PCR analysis. Table S2. Plant primers for PCR analysis. [file 12951_2023_1835_MOESM1_ESM.docx]

**1. Supplementary Figures and Legends**

**
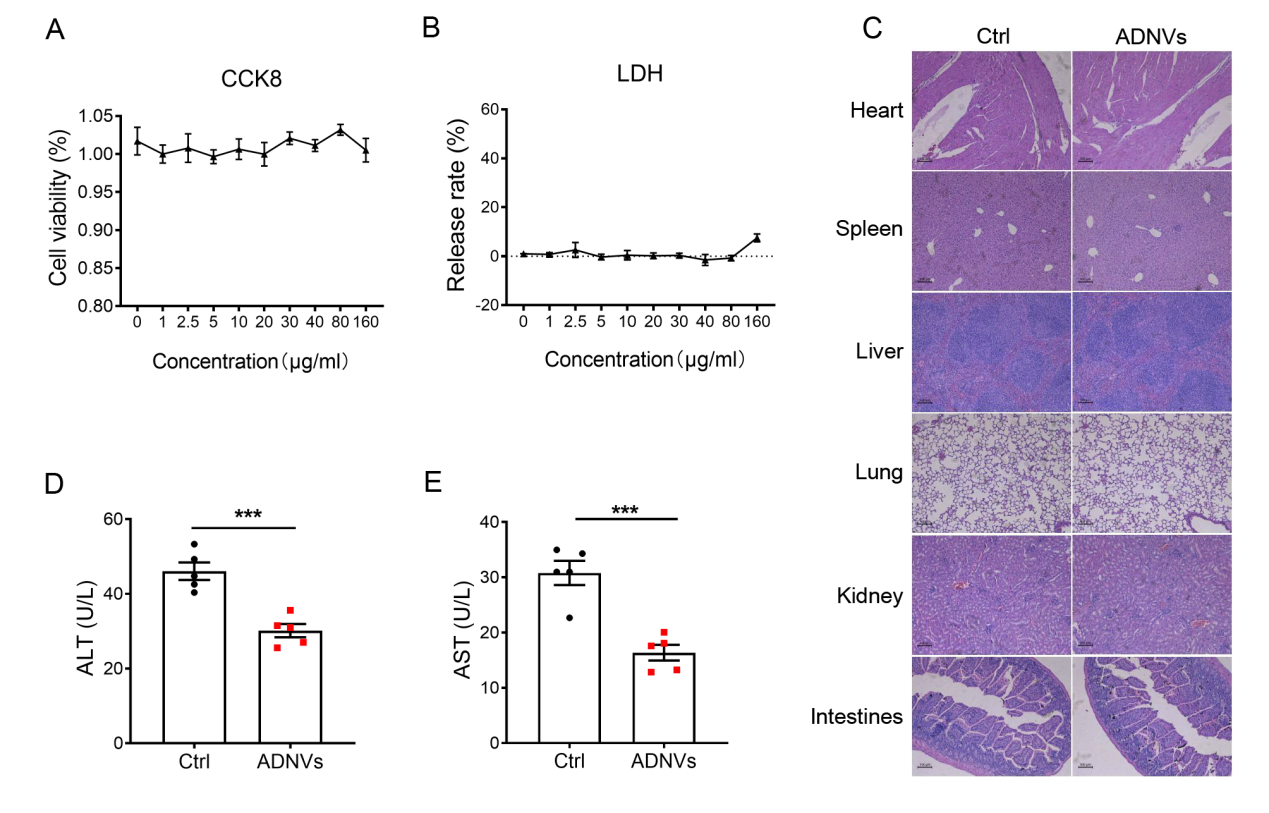
**

**Figure S1. Analysis of biocompatibility of ADNVs.**

(A, B) The impact of ADNVs on the survival of BMDMs as analyzed by CCK-8 and LDH assays. (C) Histological analysis of the main organs, and (D, E) Serum levels of ALT and AST in mice treated with ADNVs (25 mg/kg, i.p.) or vehicle. ****p* < 0.001 (Student’s t-test). Scale bar = 100 μm. The results are from one of two independent experiments. Representative images are shown and the data are presented as means ± SEM.


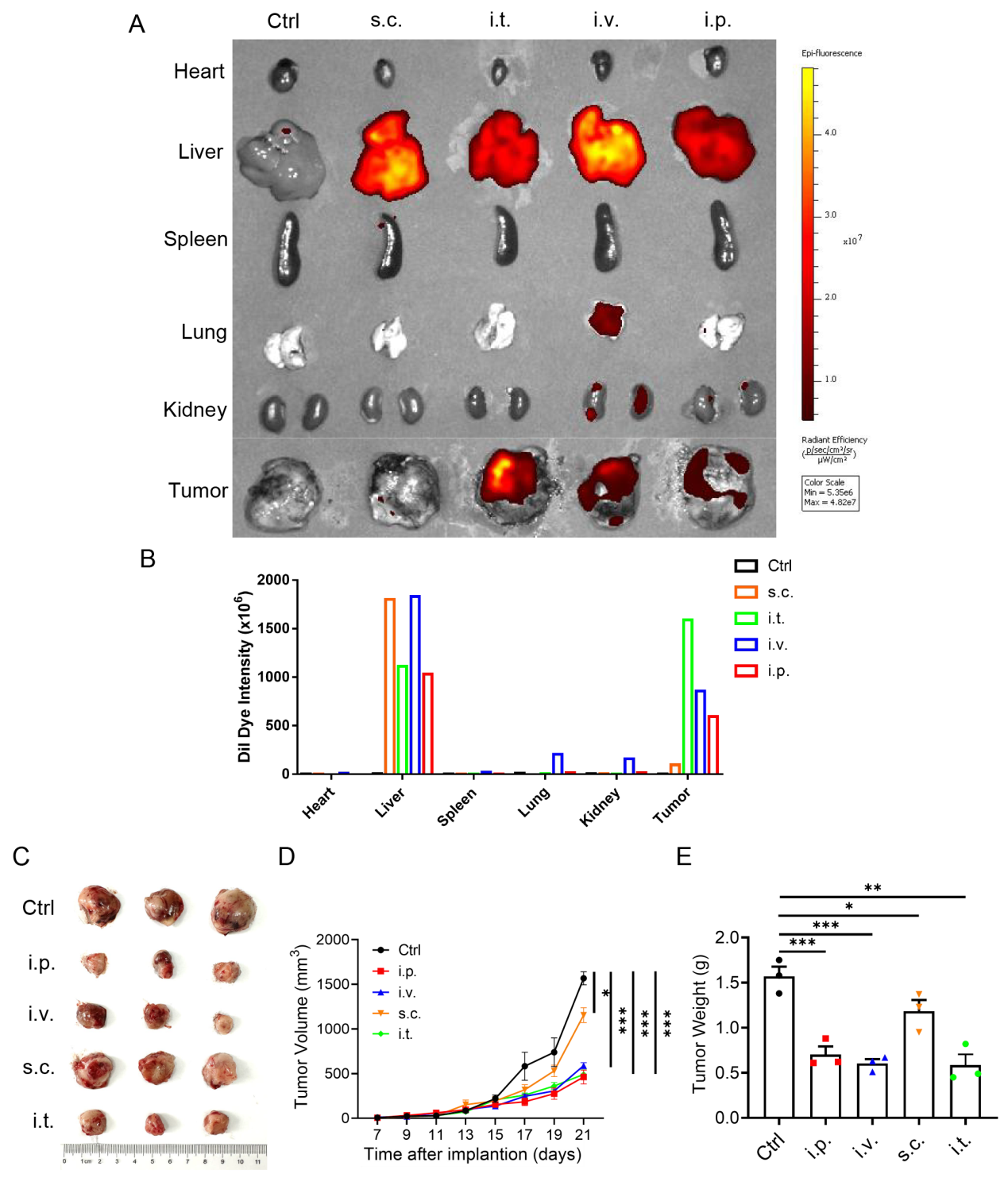


**Figure S2. Effects of different injection routes on biodistribution and tumor control of ADNVs *in vivo*.**

(A) *In vivo* biodistribution of Dil-labelled ADNVs administrated intraperitoneal (i.p.), intravenous (i.v.), subcutaneous (s.c.) and intratumoral (i.t.) to tumor-bearing mice for 24 h. (B) Corresponding quantification of accumulated fluorescence signals in different organs. (C-E) C57BL/6 mice (n = 3) were implanted with LLC cells for 7 d, and then treated with ADNVs (25 mg/kg, i.p., i.v., s.c. and i.t.) once every 3 d for a successive 2 week. Mice were sacrificed and tumors were collected at day 21. (C) Gross photos of tumors at the end of experiments. (D) Tumor growth profiles in tumor-bearing mice. The data represent mean ± SEM. **p* < 0.05, ****p* < 0.001. Two-way ANOVA and Bonferroni post-tests were used for comparison. (E) Tumor weights at the end of the experiment. The data represent mean ± SEM. **p* < 0.05, ***p* < 0.01, ****p* < 0.001. One-way ANOVA and Tukey’s significant difference *post hoc* test was used for statistical comparison.


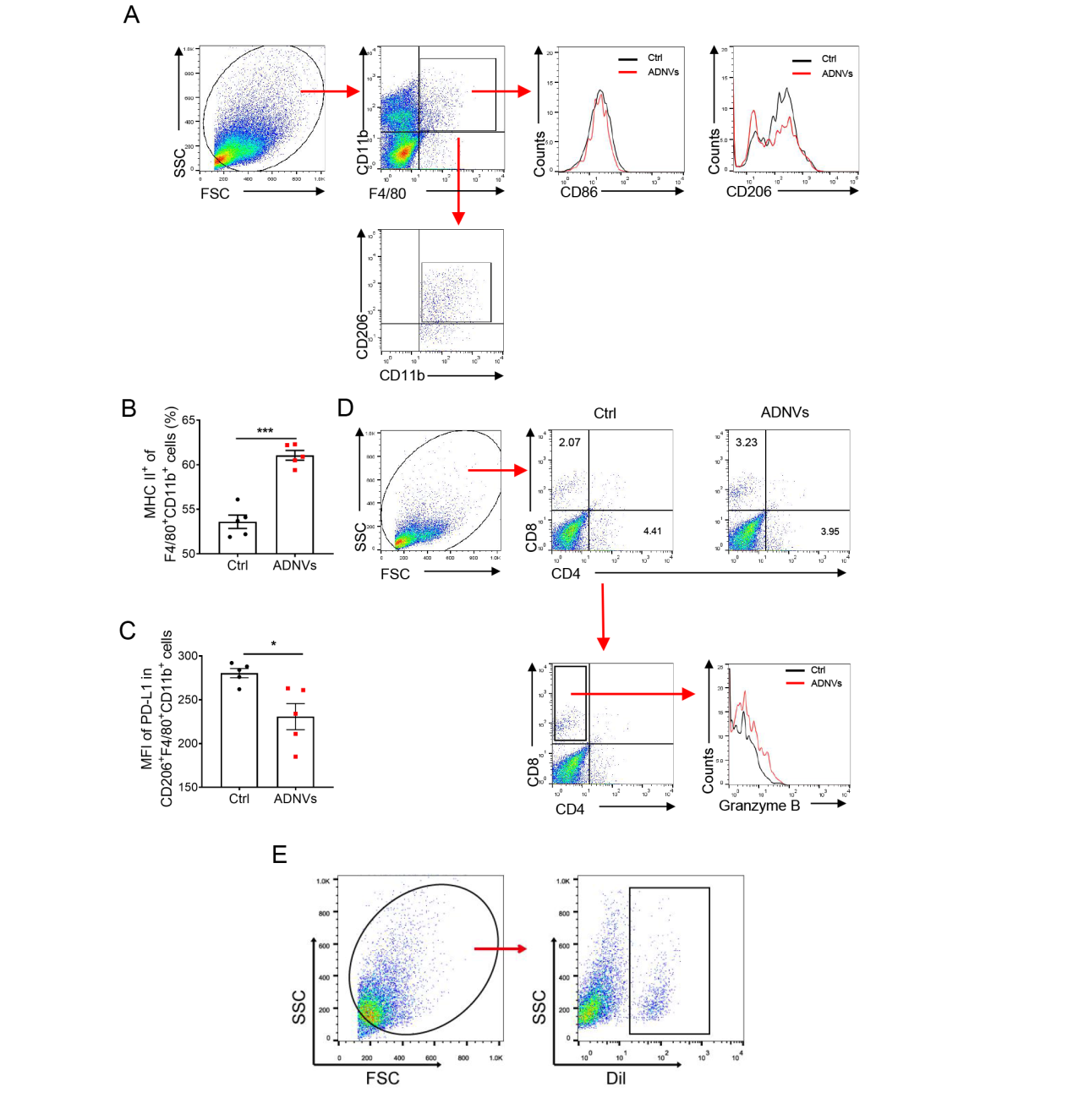


**Figure S3. ADNVs promote TAMs polarization towards M1 phenotype.**

C57BL/6 mice were implanted with LLC cells for 7 d, and then treated with ADNVs (25 mg/kg, i.p.) or vehicle every 3 d for a successive 2 week. Mice were sacrificed and tumors were collected at day 21. (A) Gate strategy for flow cytometry of macrophage subsets. (B, C) Flow cytometry of MHC Ⅱ^+^ or PD-L1^+^CD206^+^ macrophage subset. **p* < 0.05, ****p* < 0.001 (Student’s t-test). (D) Gate strategy for flow cytometry of CD4^+^ or CD8^+^ T cells. (E) Gate strategy for flow cytometry of Dil^+^ cells. Representative images are shown and the data from one of two experiments are presented as means ± SEM.


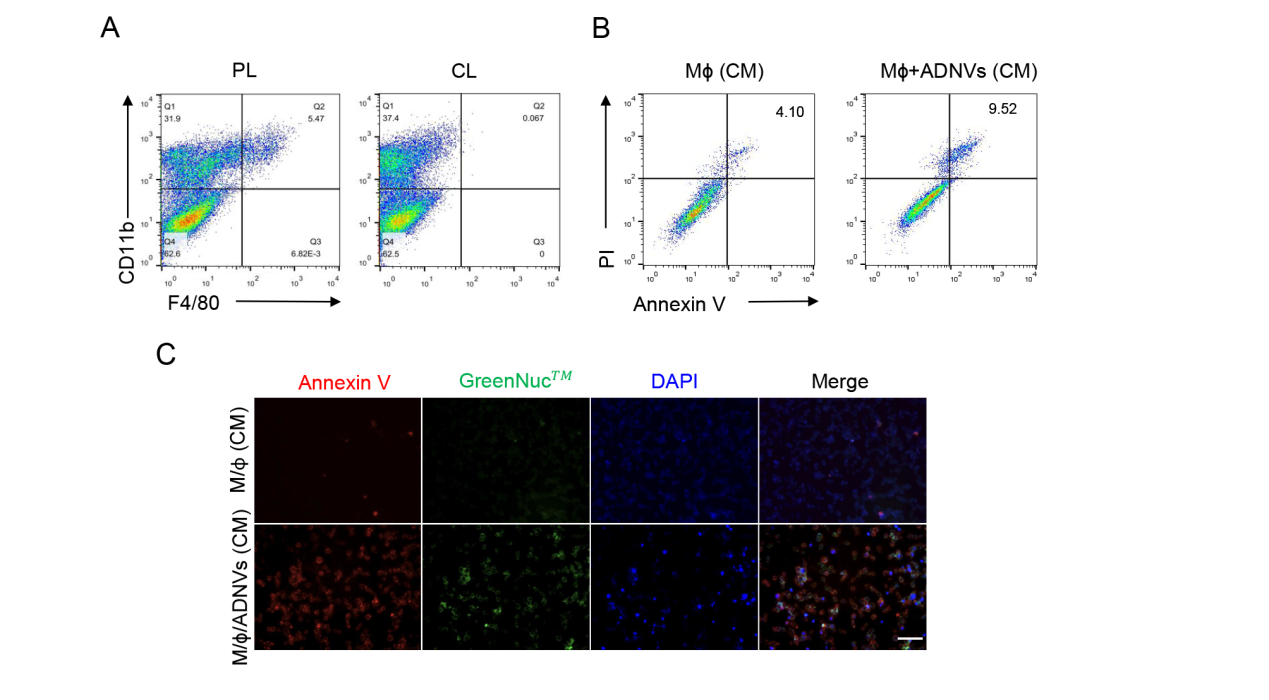


**Figure S4. ADNVs act through TAMs to exert the anti-tumor effect.**

(A) C57BL/6J mice were implanted with LLC cells for 7 d and then inoculated with CL or PL every 4 days to deplete TAMs. The depletion efficacy was confirmed by flow cytometry of CD11b^+^F4/80^+^ macrophages in tumors at day 21. (B, C) M2-polarized macrophages were incubated with or without ADNVs for 24 h. The culture medium were collected and applied to LLC cells. Flow cytometery of apoptotic cells by Annexin V/PI staining (B); Immunofluorescence staining of apoptotic cells with Annexin V/caspase-3 (C). Scale bar = 50 μm. Nuclei, DAPI. Shown are representative images from one of two experiments.


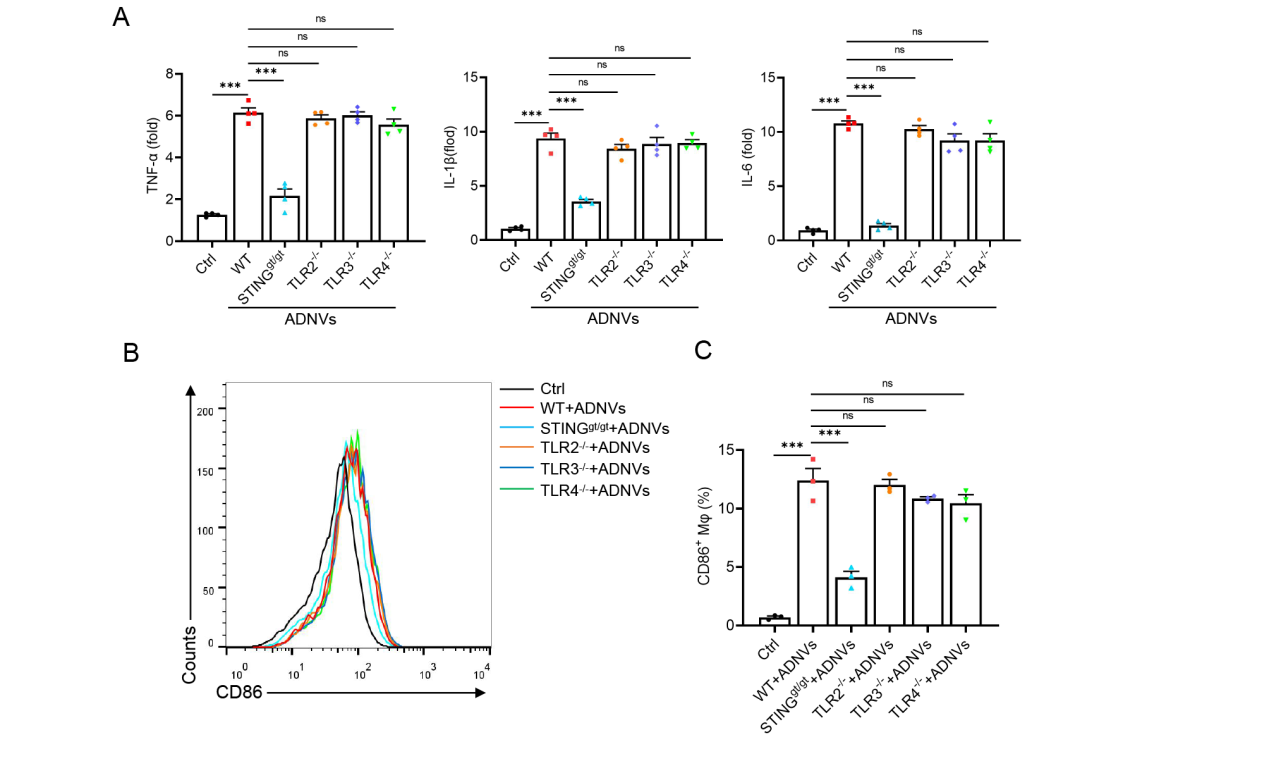


**Figure S5: The immunoregulatory function of ADNVs is not through TLR2, TLR3, or TLR4 pathway.**

(A) M2 macrophages were prepared from WT, STING^gt/gt^, TLR2^-/-^, TLR3^-/-^ and TLR4^-/-^ mouse BMDMs by stimulated with IL-4, and then treated with ADNVs (20 μg/mL) for 24h. qRT-PCR analysis of the expression of TNF-α, IL-1β and IL-6 genes. (B, C) Flow cytometry analysis of M1 marker (CD86) expression on BMDMs. (A-C) ****p* < 0.001, ns: not significant. One-way ANOVA and Tukey’s significant difference *post hoc* test was used for statistical comparison. The data are from one of two independent experiments, and represent as mean ± SEM.

**2. Supplementary tables**

**Tablse S1 Mouse primers for quantitative RT-PCR analysis**

| Name | | Sequence (5′→3′) |
| --- | --- | --- |
| *β-actin* | Forward primer | CATGTACGTTGCTATCCAGGC |
|  | Reverse primer | CTCCTTAATGTCACGCACGAT |
| *TNF-α* | Forward primer | AAGCCTGTAGCCCACGTCGTA |
|  | Reverse primer | GGCACCACTAGTTGGTTGTCTTTG |
| *IL-1β* | Forward primer | TAGGGAAAAATTTAGGGCCAGAT |
|  | Reverse primer | TCCAGGATGAGGACATGAGCAC |
| *IL-6* | Forward primer | GAGGATACCACTCCCAACAGACC |
|  | Reverse primer | AAGTGCATCATCGTTGTTCATACA |
| *IL-10* | Forward primer | GACCAGCTGGACAACATACTGCTAA |
|  | Reverse primer | GATAAGGCTTGGCAACCCAAGTAA |
| *iNOS* | Forward primer | GGAGCGAGTTGTGGATTGTC |
|  | Reverse primer | GTGAGGGCTTGGCTGAGTGAG |
| *Arg1* | Forward primer | CAGAAGAATGGAAGAGTCAG |
|  | Reverse primer | CAGATATGCAGGGAGTCACC |
| *CD86* | Forward primer | TGTTTCCGTGGAGACGCAAG |
|  | Reverse primer | TTGAGCCTTTGTAAATGGGCA |
| *CD206* | Forward primer | CTCTGTTCAGCTATTGGACGC |
|  | Reverse primer | CGGAATTTCTGGGATTCAGCTTC |

**Table S2 Plant primers for PCR analysis**

| Name | | Sequence (5′→3′) |
| --- | --- | --- |
| *COX3* | Forward primer | GTAGATCCAAGTCCATGGCCT |
|  | Reverse primer | GCATGATGGGCCCAAGTTACGGC |
| COX2 | Forward primer | TCCCACAAAGGATTGTTCATGG |
|  | Reverse primer | CCTAACTCTTACCACGTTATAT |
| *rbcL* | Forward primer | TGGACTGATGGACTTACCAGTCTTGATCG |
|  | Reverse primer | ACTTCGCAAGCAGCAGCTAATTCAGGACT |
